# All-Perovskite Multicomponent Nanocrystal Superlattices

**DOI:** 10.1021/acsnano.3c13062

**Published:** 2024-03-06

**Authors:** Taras V. Sekh, Ihor Cherniukh, Etsuki Kobiyama, Thomas J. Sheehan, Andreas Manoli, Chenglian Zhu, Modestos Athanasiou, Marios Sergides, Oleksandra Ortikova, Marta D. Rossell, Federica Bertolotti, Antonietta Guagliardi, Norberto Masciocchi, Rolf Erni, Andreas Othonos, Grigorios Itskos, William A. Tisdale, Thilo Stöferle, Gabriele Rainò, Maryna I. Bodnarchuk, Maksym V. Kovalenko

**Affiliations:** † Institute of Inorganic Chemistry, Department of Chemistry and Applied Biosciences, ETH Zürich, 8093 Zürich, Switzerland; ‡ Laboratory for Thin Films and Photovoltaics, Empa−Swiss Federal Laboratories for Materials Science and Technology, 8600 Dübendorf, Switzerland; § IBM Research Europe−Zürich, Rüschlikon CH-8803, Switzerland; ∥ Department of Chemical Engineering, Massachusetts Institute of Technology, Cambridge, Massachusetts 02139, United States; ⊥ Experimental Condensed Matter Physics Laboratory, Department of Physics, University of Cyprus, 1678 Nicosia, Cyprus; # Laboratory of Ultrafast Science, Department of Physics, University of Cyprus, Nicosia 1678, Cyprus; ○ Electron Microscopy Center, Empa−Swiss Federal Laboratories for Materials Science and Technology, CH-8600 Dübendorf, Switzerland; □ Department of Science and High Technology and To.Sca.Lab, University of Insubria, via Valleggio 11, 22100 Como, Italy; △ Istituto di Cristallografia and To.Sca.Lab, Consiglio Nazionale delle Ricerche, via Valleggio 11, 22100 Como, Italy

**Keywords:** nanocrystals, lead halide perovskites, superlattices, nanocrystal coupling, energy transfer, exciton diffusion, superfluorescence

## Abstract

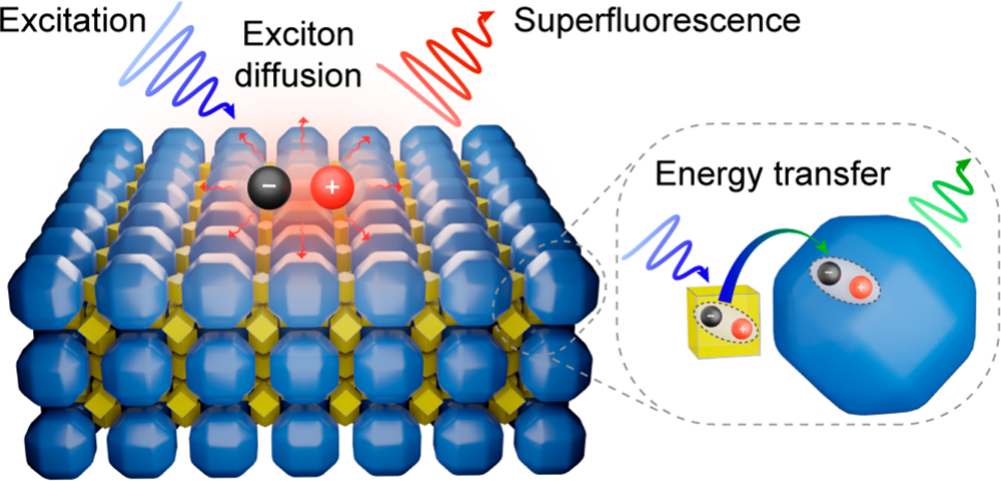

Nanocrystal superlattices (NC SLs) have long been sought as promising metamaterials, with nanoscale-engineered properties arising from collective and synergistic effects among the constituent building blocks. Lead halide perovskite (LHP) NCs come across as outstanding candidates for SL design, as they demonstrate collective light emission, known as superfluorescence, in single- and multicomponent SLs. Thus far, LHP NCs have only been assembled in single-component SLs or coassembled with dielectric NC building blocks acting solely as spacers between luminescent NCs. Here, we report the formation of multicomponent LHP NC-only SLs, i.e., using only CsPbBr_3_ NCs of different sizes as building blocks. The structural diversity of the obtained SLs encompasses the ABO_6_, ABO_3_, and NaCl structure types, all of which contain orientationally and positionally locked NCs. For the selected model system, the ABO_6_-type SL, we observed efficient NC coupling and Förster-like energy transfer from strongly confined 5.3 nm CsPbBr_3_ NCs to weakly confined 17.6 nm CsPbBr_3_ NCs, along with characteristic superfluorescence features at cryogenic temperatures. Spatiotemporal exciton dynamics measurements reveal that binary SLs exhibit enhanced exciton diffusivity compared to single-component NC assemblies across the entire temperature range (from 5 to 298 K). The observed coherent and incoherent NC coupling and controllable excitonic transport within the solid NC SLs hold promise for applications in quantum optoelectronic devices.

In recent decades, nanocrystal superlattices (NC SLs) have drawn much research interest in anticipation of collective properties, tuned by the SL structure, NC chemical composition, and morphology.^1,2^ The close proximity of NCs with long-range positional and orientational ordering may facilitate the emergence of diverse synergistic and collective effects, significantly different from ensemble-averaged properties. Specific examples include magnetic exchange coupling^3^ and dipolar interactions^4^ in SLs of magnetic NCs, conductivity enhancement in annealed PbTe-Ag_2_Te NC SLs,^5^ collective plasmonic response in noble metal-containing SLs,^6−8^ and enhanced catalytic activity,^9^ as well as improved mechanical properties.^10^ Another prominent collective effect is the emergence of the band-like transport in the semiconductor NC solids,^11,12^ albeit without firm attribution of this property to arise necessarily from the long-range NC order.

In the realm of excitonic NCs, the first occurrence of collective properties has been manifested in 3D SL assemblies of lead halide perovskite (LHP) NCs,^13−15^ seen as collective light emission, known as superfluorescence (SF).^16^ SF was previously demonstrated only in a limited number of systems such as gaseous HF,^17^ and solid-state InGaAs quantum wells.^18^ The key attributes of LHP NCs enabling SF include fast radiative rates and high oscillator strength of bright triplet excitons as the main recombination channel at low temperatures^19,20^ and slow exciton dephasing time,^21−23^ allowing the buildup of multi-NC coherence.^16,21^ The observation of SF in LHP NC SLs triggered new experiments capable of delineating its fundamental attributes and new structure–property relations. Single-component SLs with NC building blocks in the strong confinement regime were theoretically modeled based on well-known non-Hermitian radiative Hamiltonians, with predictions of 4 orders of magnitude enhancement in superradiant response.^24^ However, due to the increasing energetic disorder in the strong quantum confinement regime, the increased exciton–phonon coupling^25,26^ with accelerated dephasing time and the reduced oscillator strength, single-component SLs assembled from ∼5 nm CsPbBr_3_ NCs did not meet theoretical expectations and did not exhibit spectral features of SF.^27,28^ To further explore the rich physics behind the coherent NC coupling in LHP NC SLs, we then ventured into the exploration of binary SLs, with the aim of systematically tuning the geometrical orientation of the constituent NCs thus altering the dipole–dipole interaction. A plethora of multicomponent SLs was devised by combining 5–8.5 nm CsPbBr_3_ NCs with larger optically inactive dielectric NCs.^27,29,30^ The distinctive cubic shape of LHP NCs and the associated ligand deformations enabled the formation of dense structures exhibiting a high degree of orientational NC ordering. We could then resolve the structure–function relationship, proving that SF is highly sensitive to the LHP NC volume fraction in binary SLs. SLs of the perovskite ABO_3_-type exhibited the most efficient coupling owing to the much reduced LHP NC–NC spacing, while NaCl-type SLs did not sustain SF and coherent coupling, presumably due to much reduced LHP NC fraction.^29^

Further engineering of optically enhanced SLs comprising LHP NCs may, in principle, undertake two distinct avenues by replacing the dielectric NC component, used as rather passive dielectric spacers in the aforementioned binary SLs, with either the plasmonic or excitonic counterparts. Distance-dependent quenching or enhancement of the semiconductor NC luminescence in the proximity of a plasmonic NC^31,32^ will merit an independent study with LHP NCs as emitters. In this work, instead, we focus on all-excitonic and all-LHP NC SLs, comprising large LHP NCs (∼18 nm, i.e., in the weak confinement regime) and LHP NCs in strong and moderate confinement regime, i.e., of the same size-range as in our previous works (5–8.5 nm).^27,29,30^ Such choice of materials was motivated by the high emissivity of LHP NCs maintained over the large NC size range of 5–30 nm and broad temperature range (from room temperature to cryogenic temperatures),^20^ along with their facile synthesis with a high degree of size and shape-uniformity.^13,33−36^ We also acknowledge the prior-art on binary all-semiconductor NC SLs comprising conventional materials (CdSe,^37−40^ PbS,^41,42^ and PbSe^43−45^ NCs), which, however, chiefly focused on structural and thermodynamics aspects of NC self-assembly. For the binary SLs comprising PbSe NCs of two different sizes, transient absorption spectroscopy gave evidence for the directional charge transfer from the larger to the smaller bandgap NCs,^43^ without precisely identifying the specific mechanism (e.g., direct charge transfer, Förster or Dexter energy transfer).

We discovered a plethora of all-perovskite NC multicomponent SLs obtained via the coassembly of different-sized LHP NCs. Strongly confined 5.3 nm cubic-shaped CsPbBr_3_ NCs coassemble with weakly confined 17.6 nm rhombicuboctahedral CsPbBr_3_ NCs into ABO_6_- or NaCl-type SLs, depending on the particle number ratio. The increase in the size of small-component NCs up to 8.0 nm leads to the formation of perovskite-type ABO_3_- or NaCl-type SLs. Delving into the emerging optical properties within these metamaterials, we demonstrated efficient energy transfer from strongly confined CsPbBr_3_ NCs to weakly confined CsPbBr_3_ NCs in ABO_6_-type SLs, at both room and cryogenic temperature. Energy funneling was evidenced by ultrafast pump–probe spectroscopy, featuring a faster decay rate for strongly confined NCs, acting as the energy donor, in binary SL compared to the single-component sample, and a longer rise time for the weakly confined NCs, acting as the energy acceptor, suggesting the occurrence of Förster-like energy transfer. The characteristic SF features, radiative lifetime shortening and ringing behavior, are displayed by single-component 17.6 nm CsPbBr_3_ SL as well as ABO_6_-type all-perovskite NC SL in time-resolved PL spectra at a high excitation regime. Exciton diffusion studies revealed enhanced exciton diffusivity of 0.064 cm^2^/s in binary SLs compared to single-component SLs (0.025 cm^2^/s and 0.028 cm^2^/s for 5.3 and 17.6 nm CsPbBr_3_ NCs, respectively) at cryogenic temperatures, affirming the critical role played by the constituent NC building blocks and the high degree of structural order.

## Results and Discussion

### Colloidal NC Self-Assembly

A high degree of SL ordering and extended SL domain sizes (several μm) are essential technical prerequisites for studying the structural-optical property relationships in NC SLs. Such attributes can be accomplished via the bottom-up self-assembly of colloidal NCs (by the slow evaporation method)^1^ employed in the present work for the SL preparation. Self-assembly of sterically stabilized colloidal NCs is typically rationalized as an interaction of hard spheres (shapes), driven by the total entropy maximization,^1,46^ yielding densely packed structures, i.e., face-centered cubic (*fcc*) or hexagonal close-packed (*hcp*) lattices for single-component systems of spheres, exemplified by gem opals^47^ and colloidal beads.^48^ At a late stage of solvent evaporation, NC ordering maximizes translational and rotational entropies as an additive quantity over the NC ensemble, compensating for the loss in the configurational entropy. There also exists a general consensus that the self-assembly of sub-20 nm colloidal NCs capped with hydrocarbon-based ligands (1–2 nm in length) is a more complex interplay between the entropic and enthalpic contributions.^39,42,49,50^ Ligand softness and deformability^51−54^ explain a greater diversity of observed structures already in single-component^28,55^ and binary SLs.^56^ When coassembling two kinds of spherical NCs, two experimental variables are typically adjusted: the effective size ratio between two particles γ_eff_ (γ_eff_ = *r*_A_/*r*_B_, *r*_A_, *r*_B_ being effective particles’ radii) and particle number ratio. The structural space of the obtained binary SLs goes well beyond the three densest hard-sphere structures (AlB_2_, NaCl, and NaZn_13_),^40,57^ totaling to several dozens of binary,^58−60^ ternary,^29,61,62^ and quasicrystalline^63,64^ SLs. NC shape anisotropy provided a gateway to new SL structures otherwise inaccessible upon the coassembly of spherical-only NCs. Examples include periodic lattices formed upon mixing spherical NCs with nanorods,^65^ nanoplates,^60^ nanowires,^66^ octapods,^67^ and nanocubes.^30^

LHP nanocubes were previously coassembled with spherical dielectric NaGdF_4_ NCs, resulting in a variety of SLs (ABO_3_-, ABO_6_-, NaCl-, AlB_2_-types) with large SL domains and a high degree of orientational order.^27,29,30^ High propensity to form SL structures of ABO_3_- and ABO_6_-types, uncommon for all-sphere mixtures, was shown to arise from the combined effect of the sharp cubic core of LHP NCs and ligand deformability at the NCs’ vertices and edges according to the Orbifold Topological Model (OTM).^68^ The latter affords additional SL compactness. In the footsteps of these observations, herein we extend the compositional space to the combination of two LHP NC types. Particularly, the sharp cubic shape of the small NC component and the (quasi)-spherical morphology of the other one facilitate the NC intermixing as opposed to segregation in single-component SLs.^69^

### Selection and Characterization of SL Building Blocks

**Figure 1:**
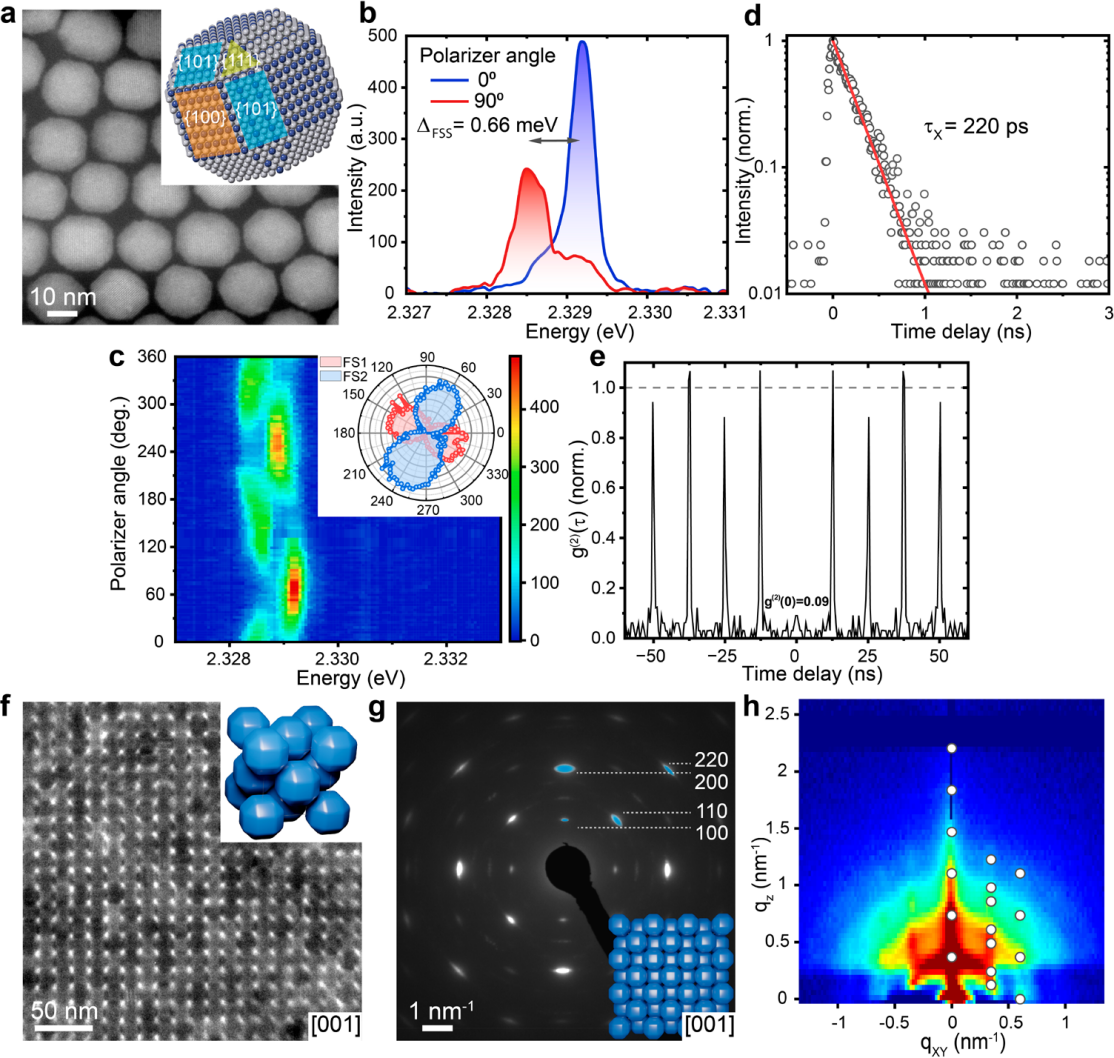
Rhombicuboctahedral CsPbBr_3_ NCs. (a) The high-angle annular dark-field scanning transmission electron microscopy (HAADF-STEM) image of a CsPbBr_3_ NC monolayer showing the NC shape and oblique packing; (inset) a structural model of rhombicuboctahedral CsPbBr_3_ NC with depicted facet types. (b) PL spectra of a single ∼16 nm CsPbBr_3_ NC at 4 K, revealing a doublet exciton fine structure at polarization angles of 0° (blue curve) and 90° (red curve). (c) The evolution of PL spectra with the rotation of a linear polarizer and (inset) the respective polar plot, showing the intensity variation of the doublet exciton fine structure. (d) PL lifetime trace of a single ∼16 nm CsPbBr_3_ NC, demonstrating ultrafast PL emission (∼220 ps in this specific NC) with a monoexponential decay over 2 orders of magnitude. (e) The *g*^(2)^(τ) trace revealing photon antibunching behavior (*g*^(2)^(0) = 0.09), which attests pure single photon emission from rhombicuboctahedral CsPbBr_3_ NCs. (f) TEM image of an *fcc*-packed single-component SL assembled from 17.6 nm CsPbBr_3_ NCs with (inset) the *fcc*-type unit cell model. (g) The corresponding WAED pattern of a single [001]_SL_-oriented SL domain with reflections from a preferential orientation marked with blue; here, and throughout this manuscript, the scale bar (in nm^–1^) of the ED images refers to the 1/*d* unit, and not to the scattering vector length *q* = 2π/d. (inset) A structural model of a [001]_SL_-oriented SL. (h) 2D GISAXS pattern of an [111]_SL_-oriented *fcc*-type CsPbBr_3_ SL, confirming the proposed packing and demonstrating both the in-plane and out-of-plane order of the SL, with theoretically predicted reflections shown as white circles.

First, it is preferable that the two NC building blocks have significantly different emission wavelengths, facilitating the emergence and observation of energy transfer or other synergistic effects.^70,71^ Second, it is imperative that two kinds of LHP NCs are of the same composition, e.g., CsPbBr_3_, to avoid complexities arising from the ion-exchange between NCs.^72,73^ At present, CsPbBr_3_ NCs are synthetically accessible as monodisperse colloids in a broad size range (5–30 nm).^13,33−36^ Third, building upon the previous experience,^27,29,30^ we set to coassemble sub-10 nm cuboid CsPbBr_3_ NCs with ∼20 nm quasi-spherical CsPbBr_3_ NCs (instead of spherical dielectric NCs) aiming (i) to emulate the shapes and size-ratios successful in the earlier studies^27,29,30^ and (ii) to maximize the bandgap energy difference. Monodisperse 5.3 and 8 nm CsPbBr_3_ NCs of cuboid shape (Figure S1) were synthesized by the hot-injection method,^13,28,33,34^ by adding Cs oleate into the mixture of PbBr_2_, oleic acid (OA), and oleylamine (OLA) in 1-octadecene (ODE). The labile OA-OLA ligand shell was replaced with shorter didodecyldimethylammonium bromide (DDAB) ligand by a postsynthetic treatment.^74,75^ NCs of both sizes, 5.3 nm, and 8.0 nm, crystallize in the orthorhombic *Pnma* space group and exhibit termination by {101} and {010} planes, which, for simplicity, are hereafter referred to as pseudocubic {100} planes.^29,76^ In the single-component SL, 8.0 nm NCs assemble into a cubic lattice,^13^ whereas 5.3 nm NCs, in line with their higher softness, arrange into a rhombic lattice with an obtuse angle of 104°.^28^ As a larger LHP NC building block, 14–18 nm CsPbBr_3_ NCs with rhombicuboctahedral shape (Figure 1a) were synthesized by adopting a hot-injection method (210–220 °C) that utilizes phenacyl bromide as a source of bromide,^35^ followed by the exchange of OLA-OA ligands with DDAB ligands. Structurally, these large NCs are terminated by 26 facets: 6, 12, and 8 for the {100}, {110}, and {111} planes, respectively (Figure 1a, inset). In a monolayer, these NCs pack in an oblique (pseudohexagonal) planar lattice (Figure 1a), manifesting their rather quasi-spherical effective shape. They typically orient with either ⟨100⟩_NC_ or ⟨110⟩_NC_ directions parallel to the zone axis, as seen by the transmission electron microscopy (TEM, Figure S2, subscript “NC” relates to the atomic lattice of the NC itself). An important property for choosing these large LHP NCs as SL constituents is their excellent emissivity; single-dot PL studies at 4 K feature narrow emission bands, with a typical fine structure splitting (Δ_FSS_) of ca. 0.66 meV (Figure 1b,c). The time-resolved PL measurement of a single ∼17 nm CsPbBr_3_ NC (Figure 1d) demonstrates an ultrafast radiative decay (∼220 ps), as a result of the occurrence of excitonic single-photon super-radiance.^20^ Despite being in the very weak confinement regime, a single large NC exhibits strong photon-antibunching behavior, i.e., single-photon emission with high purity^20,77^ (*g*^(2)^(0) of ∼0.09; Figure 1e). All of these attributes make large NCs an attractive building block for all-perovskite/all-excitonic binary NC SLs.

Due to their high size uniformity, large rhombicuboctahedral CsPbBr_3_ NCs readily coassemble into an *fcc* lattice (Figure 1f), as predicted by Monte Carlo simulations for this shape,^78^ with a high yield and average domain area of 30 μm^2^. The intense and sharp reflections in the wide-angle electron diffraction (WAED) evidence the preferential ⟨100⟩_NC_ orientation along ⟨100⟩_SL_ (Figure 1g), with the minor contribution from the ⟨110⟩_NC_ orientation along ⟨100⟩_SL_ (Figure S3, subscript “SL” indicates sets of equivalent SL directions). 2D grazing-incidence small-angle X-ray scattering (GISAXS) pattern (Figure 1h) confirms the *fcc* packing and reveals the in-plane as well as the out-of-plane order of the single-component SL assembled from 17.6 nm NCs. The derived primitive rhombohedral unit cell (21.0 nm) is well in line with the parameter of 20.7 nm determined from TEM images and is used as an effective NC size of the 17.6 nm CsPbBr_3_ NC for the calculation of an effective size ratio. Overall, the highly faceted NC morphology of rhombicuboctahedral CsPbBr_3_ NCs and their strong propensity to self-assembly, along with strong light emissivity, make them an ideal candidate as large-component NCs for coassembly with smaller CsPbBr_3_ nanocubes.

### ABO_6_-Type SL

**Figure 2:**
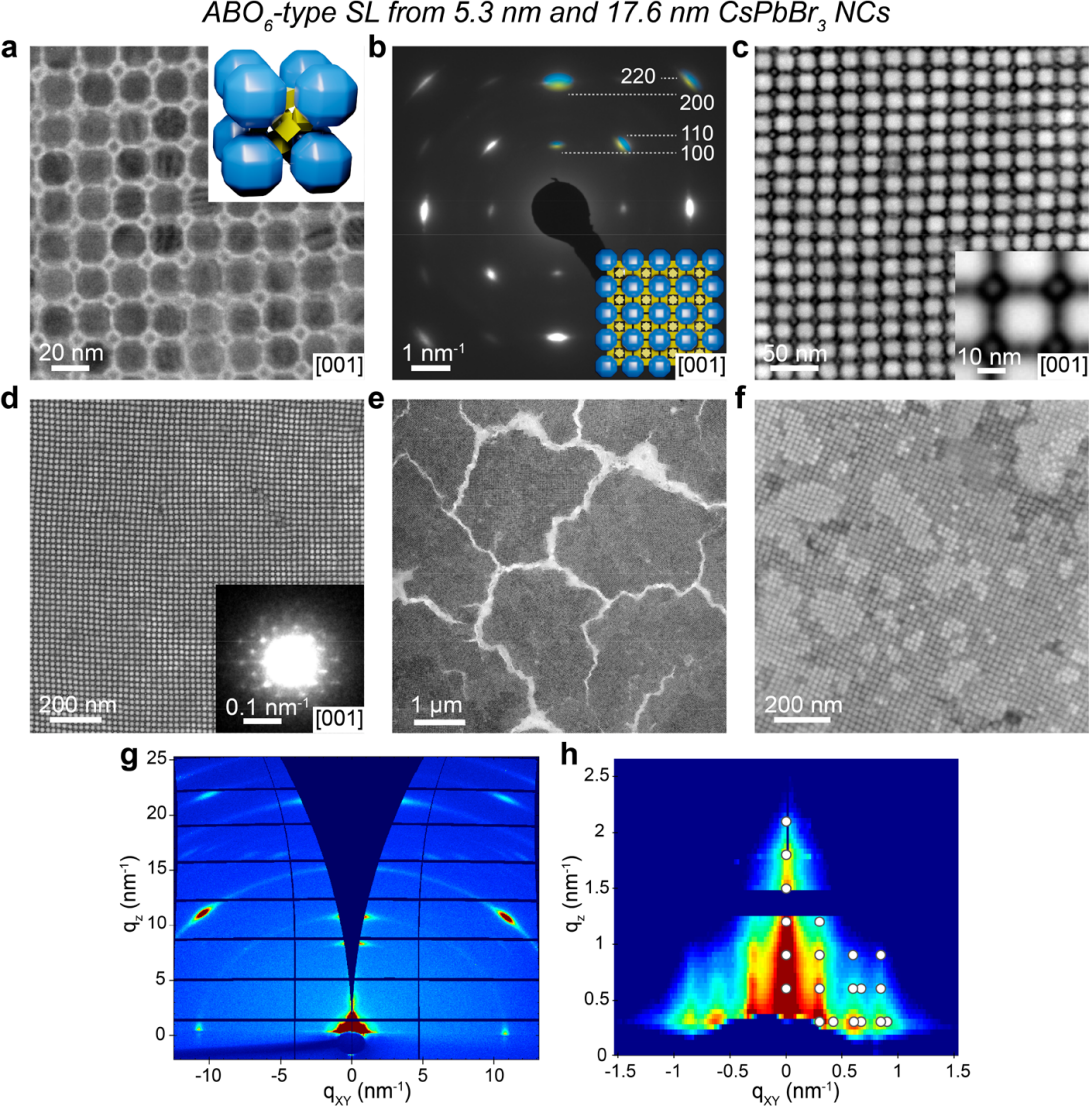
ABO_6_-type CsPbBr_3_-CsPbBr_3_ SL comprising 5.3 and 17.6 nm CsPbBr_3_ NCs. (a) TEM image of a thinner [001]_SL_-oriented ABO_6_-type domain with (inset) a structural model of a unit cell. (b) WAED pattern of a single [001]_SL_-oriented domain with the most intense reflections for 5.3 nm (B-sites) and 17.6 nm (A-sites) CsPbBr_3_ NCs marked in yellow and blue, respectively. (inset) structural model of a [001]_SL_-oriented ABO_6_-type SL. (c) HAADF-STEM image with (inset) the corresponding averaged image showing two distinct CsPbBr_3_ nanocubes in each O-site. (d) HAADF-STEM image of an ABO_6_-type SL domain with (inset) the corresponding SAED pattern. (e) Low-magnification BF-STEM and (f) SEM images revealing the domains structure and topology of the ABO_6_-type SL, respectively. (g) 2D GIWAXS and (h) GISAXS patterns of the ABO_6_-type SL fabricated on a SiN membrane. Theoretical reflections for a primitive cubic SL are indicated by white circles on the GISAXS pattern.

Among various methods for SL fabrication, the NC coassembly by solvent evaporation over a tilted substrate^42^ stands out owing to its simplicity and applicability to a variety of substrates, e.g., TEM grids or silicon nitride (SiN) membranes. Briefly, NC colloidal solutions are loaded into a tilted vial and slowly evaporated under reduced pressure to avoid far-from-equilibrium growth, enabling control over the evaporation speed and meniscus direction. First, we combined 5.3 nm CsPbBr_3_ nanocubes with 17.6 nm rhombicuboctahedral CsPbBr_3_ NCs at a high small-to-large NC number ratio (8:1), resulting in the formation of binary ABO_6_-type SL (Figure 2a). By approximating the shape of the large-component NCs to spherical, the packing density for the selected effective size ratio of constituent NCs (γ_eff_ = 0.34) exceeded that of a *fcc*-lattice within the OTM (Figure S4), explaining the prevalence of binary ABO_6_-type SL over the NC segregation into single-component SLs. The ABO_6_ structure belongs to the *Pm*3̅*m* space group (Figure 2a, inset) and can be viewed as a derivative of the ABO_3_-type SL: the A-sites are occupied by larger quasispherical CsPbBr_3_ NCs; the smaller CsPbBr_3_ nanocubes are located in the unit cell center (B-site), while each of the three O-sites is occupied by a two-cube cluster (unlike to single cubes in the ABO_3_ structure). The ⟨100⟩_NC_ directions of A- and B-sites NCs align with the ⟨100⟩_SL_, whereas two of the ⟨110⟩_NC_ of the O-site NCs coincide with ⟨100⟩_SL_. The WAED pattern (Figure 2b) predominantly displays intense 100, 110, 200, and 220 reflections originating from the larger quasi-spherical CsPbBr_3_ NCs since part of the reflections from the 5.3 nm CsPbBr_3_ nanocubes overlap with them and, due to the lower volume fraction, are much weaker in intensity. Additionally, size effects make these reflections thrice broader and thus practically undetectable. Therefore, to confirm the ABO_6_ structure type and distinguish it from the ABO_3_ lattice, we implemented a template matching of the high-angle annular dark-field scanning transmission electron microscopy image (HAADF-STEM, Figure 2c). The averaged image (Figure 2c, inset) showed the presence of two O-site cube clusters positioned along the line between larger rhombicuboctahedral NCs (Figure S5). The proposed structure is in agreement with the tomography reconstruction of the [001]_SL_-oriented domain (see Supporting Information, Video S1 and Figure S5). The obtained SLs feature a high degree of regularity (Figure 2d) and distinct domains (Figure 2e) with a uniform topology (Figure 2f). Unlike the ABO_6_-type SLs coassembled from cube-sphere mixtures,^30^ all-perovskite NC ABO_6_-type SL exhibits exclusively [001]_SL_-oriented domains. On SiN membranes, the ABO_6_-type SL domains extend over 100 μm^2^, with a typical overall surface yield of 70% (Figure S6). Complementary to WAED, a 2D grazing-incidence wide-angle X-ray scattering pattern (GIWAXS, Figure 2g) confirmed the preservation of the orthorhombic NCs structure and unveiled reflections not apparent in the WAED pattern (Figure S7). The presence of a high degree of both in-plane and out-of-plane order is manifested by the sharp reflections in the small-angle electron diffraction (SAED, Figure 2d, inset) and 2D GISAXS (Figure 2h) patterns. The reflections observed in the GISAXS measurement agree well with the calculated reflections (Figure S7), confirming the formation of primitive cubic packing.

The lattice constant value (21.2 nm) estimated from the GISAXS pattern for the ABO_6_-type SL indicates a slightly larger unit cell volume and less dense packing of 17.6 nm CsPbBr_3_ NCs, compared to the single-component 17.6 nm NC SL. This further confirms the formation of a binary SL upon combining small CsPbBr_3_ nanocubes with larger quasi-spherical CsPbBr_3_ NCs.

### NaCl-Type SL

**Figure 3:**
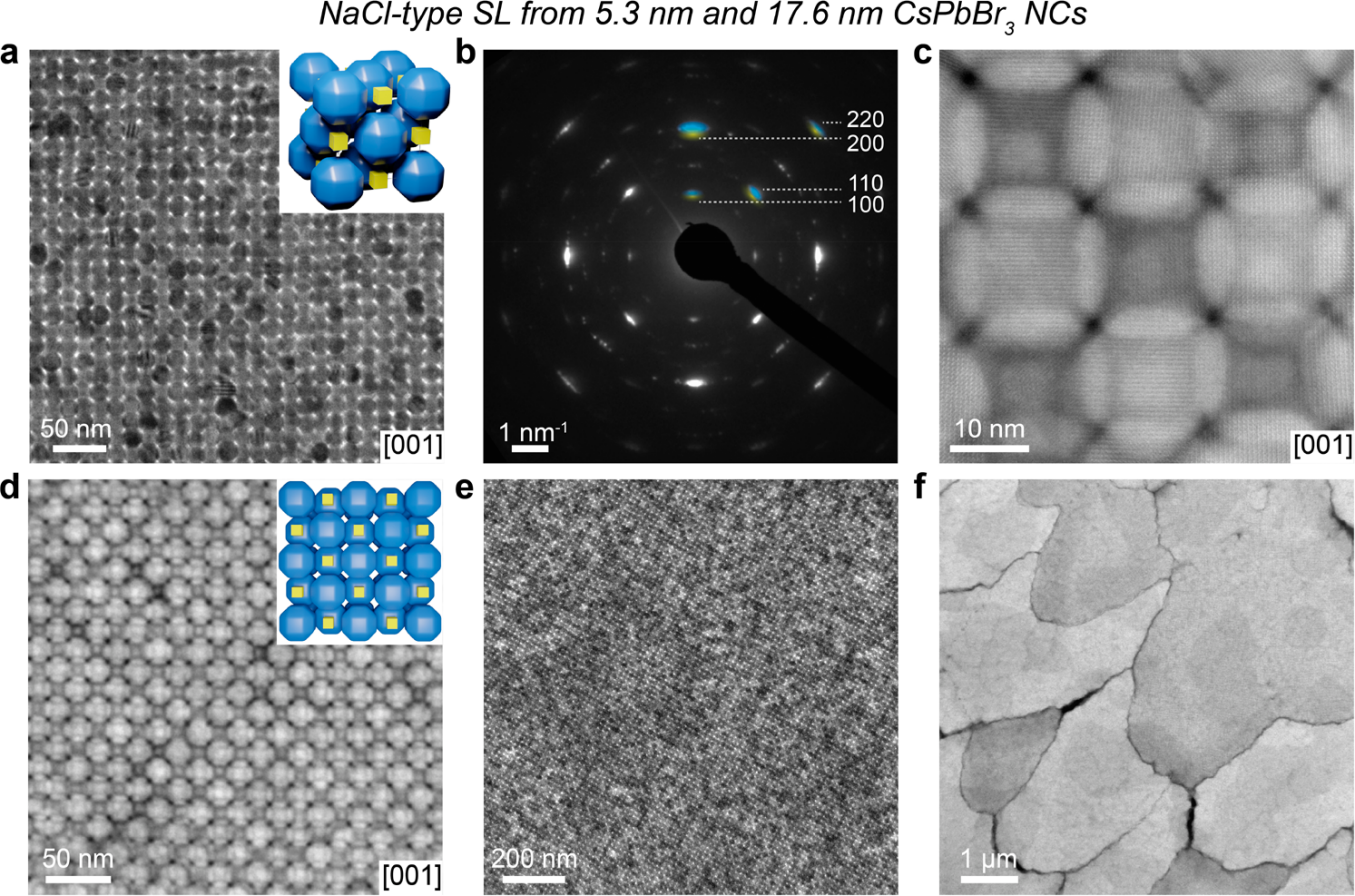
NaCl-type CsPbBr_3_-CsPbBr_3_ SL comprising 5.3 and 17.6 nm CsPbBr_3_ NCs. (a) TEM image of the [001]_SL_-oriented NaCl-type SL with (inset) the corresponding unit cell. (b) WAED pattern of [001]_SL_-oriented domain with the most intense superimposed reflections corresponding to 5.3 and 17.6 nm CsPbBr_3_ NCs marked in yellow and blue, respectively. (c) HAADF-STEM image of a small area of the NaCl-type SL domain showing the presence of small 5.3 nm CsPbBr_3_ NCs in the SL. (d) HAADF-STEM images of the [001]_SL_-oriented NaCl-type domain with (inset) the corresponding structural model containing several unit cells. Low-resolution (e) BF-STEM and (f) HAADF-STEM images of the NaCl-type SL domains illustrating the high yield of the obtained SL and typical few-μm-large domains.

In the same 5.3 + 17.6 nm NC system, a lower small-to-large NC number ratio yields a binary SL with a NaCl-type structure (space group *Fm*3̅*m*, Figure 3a). The sharp reflections in the WAED pattern of a [001]_SL_-oriented domain (Figure 3b) indicate the preferential orientation of NCs in the SL with their ⟨100⟩_NC_ or ⟨110⟩_NC_ aligned with the ⟨100⟩_SL_ featuring a higher degree of orientational order than in previously described NaCl-type SL.^30^ The WAED pattern does not differentiate different NC types in this case due to the absence of characteristic NC reflections. In HAADF-STEM, small 5.3 nm nanocubes are clearly visible under both high and low magnifications (Figure 3c,d). NaCl-type SL was obtained with a high yield, without concomitant SL types, such as ABO_6_-type SL. On SiN membranes, SL domains extend over a few μm, with high areal coverage (Figures 3e,f). The increase in the size of the small-component nanocubes to 8.5 nm leads to the formation of NaCl-type SL as well (Figure S8). Interestingly, the presence of 8.5 nm sharp nanocubes suppresses the orientational disorder of the rhombicuboctahedral 17.6 nm NCs in the *fcc* sublattice of a NaCl-type SL, as is evident from the four 110 reflections in the WAED pattern (Figure S8b) originating from a single orientation, whereas a single component *fcc*-type SL of 17.6 nm NC features eight 110 reflections from three distinct NC orientations (Figure S3).

### ABO_3_-Type SL

**Figure 4:**
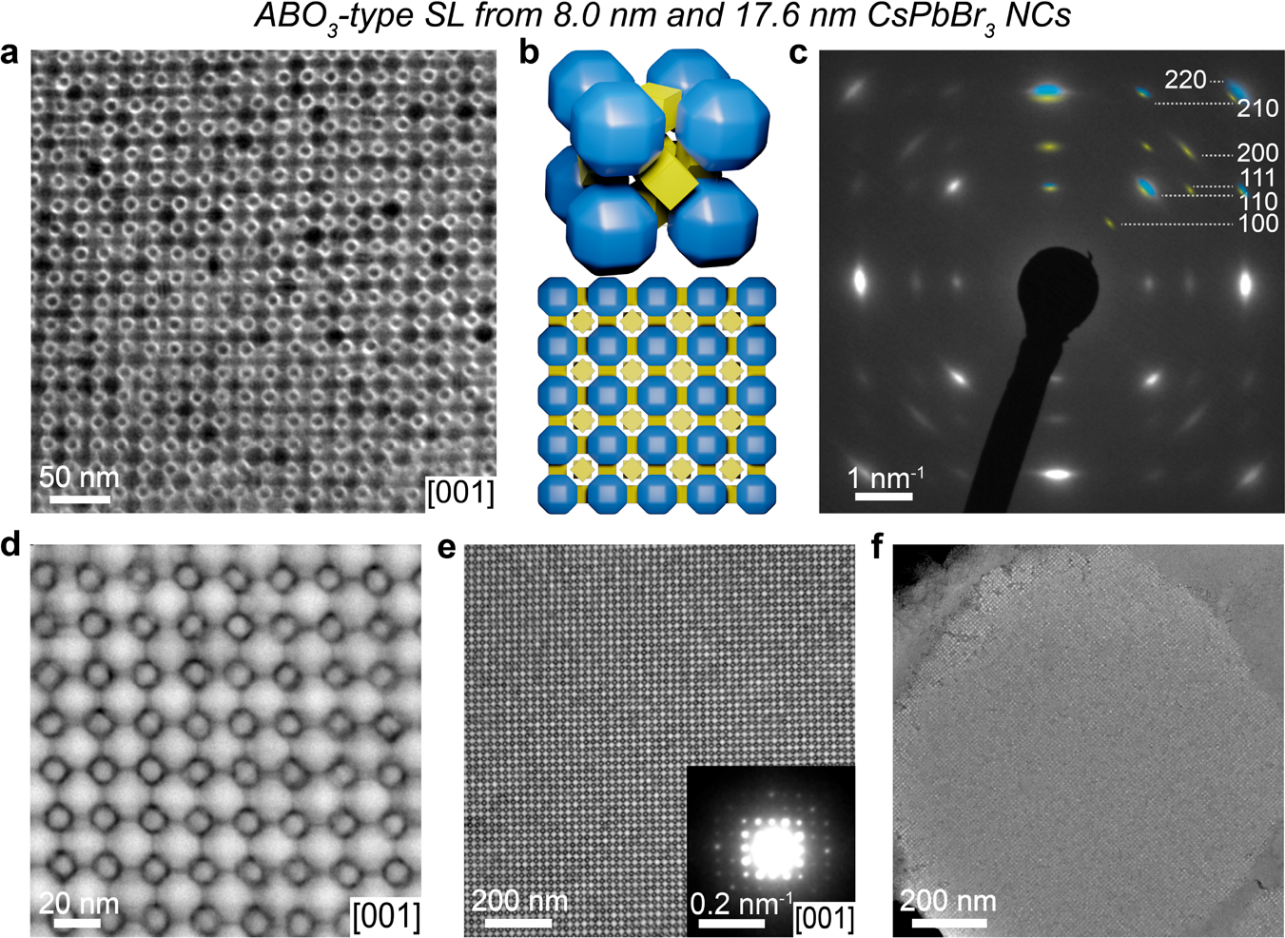
ABO_3_-type CsPbBr_3_-CsPbBr_3_ SL comprising 8.0 and 17.6 nm CsPbBr_3_ NCs. (a) TEM image of the [001]_SL_-oriented ABO_3_-type domain with (b) the corresponding unit cell and [001]_SL_-oriented ABO_3_-type SL structural models. (c) WAED pattern of the [001]_SL_-oriented ABO_3_-type SL with the most intense reflections marked for 8.0 and 17.6 nm CsPbBr_3_ NCs (yellow and blue, respectively), show distinct arcs, confirming a high degree of NC orientational ordering. (d) HAADF-STEM image of a small area within the SL domain. (e) Low magnification HAADF-STEM image and (inset) the corresponding SAED pattern with sharp reflections displaying in-plane order in the SL. (f) SEM image of the ABO_3_-type SL illustrating the domain size and uniform topology of the SL.

Co-assembly of 8.0 nm CsPbBr_3_ nanocubes with 17.6 nm CsPbBr_3_ counterparts at a high particle number ratio (5.5:1) yields an ABO_3_-type SL (Figure 4a), which also belongs to the *Pm*3̅*m* space group. For the chosen size ratio (γ_eff_ ≈ 0.5), the ABO_3_-type SL is denser compared to the competing *fcc* packing of spherical NCs (Figure S4).

Similar to the ABO_6_ structure, the A-sites in the ABO_3_ structure are occupied by larger quasi-spherical NCs (Figure 4b), while smaller nanocubes reside at the unit cell center (B-site) and on the faces (O-site). The WAED pattern (Figure 4c) contains intense reflections originating from 17.6 nm A-site NCs, indicating the preferential orientation of 17.6 nm NCs with their ⟨100⟩_NC_ directions along ⟨100⟩_SL_. This differs from the cube-sphere coassembly into ABO_3_-type SL, where spherical NCs are not orientationally locked, leading to the appearance of rings in the WAED pattern.^29^ 8.0 nm NCs are also well-oriented, with the B-site cubes aligned in the same direction as the A-site NCs. The O-site cubes exhibit an orientation where two of their ⟨110⟩_NC_ directions coincide with the ⟨100⟩_SL_, as confirmed by the presence of characteristic 110 and 111 reflections in the WAED pattern. Therefore, the 17.6 nm A-site and 8.0 nm O-site NCs primarily contact each other via their {110} and {100} facets, respectively. As evident by HAADF-STEM (Figure 4d), the NC columns are well-resolved and correspond to the proposed SL structure. The few μm-sized ABO_3_-type domains are laterally regular (Figure 4e), ordered in the in-plane direction (Figure 4e, inset), and exhibit a uniform topology (Figure 4f). An ABO_3_-type SL was also observed by combining smaller quasi-spherical 14.2 nm CsPbBr_3_ NCs with 8.5 nm CsPbBr_3_ nanocubes (Figure S9). On the other hand, similarly sized all-cuboid systems did not yield binary SLs, underlining the importance of a quasi-spherical shape of the A-site component for SL formation (Figure S10).

### Carrier Dynamics by Ultrafast Spectroscopy

**Figure 5:**
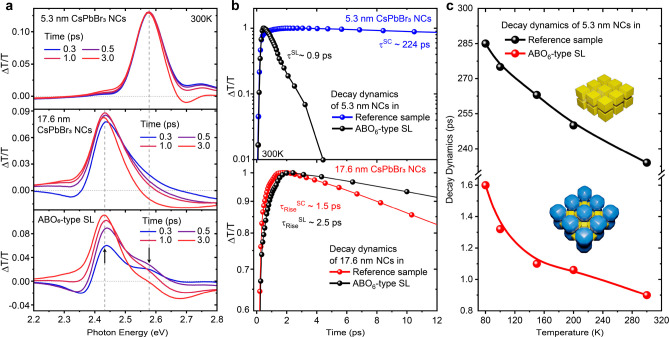
Probing ultrafast energy transfer in all-perovskite SLs by pump–probe spectroscopy. (a) Representative time sequence of spectra at early pump–probe delay times from 5.3 nm CsPbBr_3_ NCs, 17.6 nm CsPbBr_3_ NCs and ABO_6_-type SL measured at 300 K. (b) Decay dynamics of the 5.3 and 17.6 nm CsPbBr_3_ NCs in the reference samples and in a binary ABO_6_-type SL. The decay time of 5.3 nm CsPbBr_3_ NCs is much faster in the SL, while 17.6 nm CsPbBr_3_ NCs exhibit a rise time delay indicating an efficient energy transfer from 5.3 to 17.6 nm CsPbBr_3_ NCs. (c) Exciton bleaching band decay times of the 5.3 nm NCs in the reference sample and binary ABO_6_-type SL as a function of temperature in the 80 to 300 K range.

Detailed optical studies were conducted on ABO_6_-type SLs (5.3 + 17.6 nm NCs) due to the large difference in PL peak emission (∼25 nm) between the constituent NCs, a higher fraction of small-component NCs compared to NaCl-type SL and large, phase-pure SL domains. Small NCs can act as donors transferring absorbed energy to larger NCs, enabling Förster-like energy transfer in highly ordered mesoscale systems. The exciton dynamics at room temperature were first examined by pump–probe differential transmission spectroscopy. Figure 5a presents a sequence of representative differential transmission spectra from a binary SL and the reference 5.3 and 17.6 nm NC samples, within the range of 0.3–3 ps after the pump pulse. In the reference NC samples, the spectra are dominated by bleaching bands centered at 480 and 508 nm attributed to the respective 5.3 and 17.6 nm NC ground state excitons, while the SL spectra contain contributions from the bleaching bands of both NC types. The spectra of reference samples vary negligibly during the first 0.3–3 ps, because the exciton recombination in both 5.3 and 17.6 nm NC samples occurs at substantially longer time scales, as is evident from Figure 5b, depicting the decay dynamics of 5.3 and 17.6 nm CsPbBr_3_ NCs in reference and binary SL samples. In contrast, the respective features from the SL exhibit a substantially larger variation across the same temporal range, characterized by a fast quenching of the 5.3 nm NC (donor) band and a slow rise of the 17.6 nm NC (acceptor) bleaching band. Exciton lifetime, probed at the energy of small NCs (donor), is on the order of a few picoseconds in the ABO_6_-type SL, i.e., much shorter compared to the exciton relaxation time found in the reference 5.3 nm NC sample (224 ps). The fast band depletion is attributed to an efficient energy transfer from the higher energy 5.3 nm NCs, acting as donors, to the lower energy 17.6 nm NC energy acceptors.

When the bleaching band dynamics of 17.6 nm NCs are probed in the reference and the binary SL sample (Figure 5b), a rise time delay on the order of 1 ps is seen in the binary SL compared with the pristine NC sample. Such delayed excitation of the NCs indicates that besides a direct ultrafast pumping by the femtosecond laser, an additional, slower excitation channel exists, providing direct evidence for the energy flow from 5.3 nm NCs to 17.6 nm NCs. The exciton lifetime of 5.3 nm NCs in the SL and reference samples was also monitored as a function of temperature (Figure 5c). The accelerated exciton decay of 5.3 nm NCs in the binary SL probed through the 80 to 300 K range, indicates that the energy transfer process remains highly efficient over a wide range of temperatures.

The occurrence of the energy transfer is further corroborated by time-resolved PL spectroscopy (Figures S11 and S12) and photoluminescence excitation spectroscopy (PLE, Figure S13) at cryogenic temperatures. When the PL spectrum is plotted on the logarithmic scale, the residual PL from 5.3 nm NCs is observed (Figure S13), albeit with 2 orders of magnitude lower intensity due to energy transfer. The PLE is obtained by monitoring the emission peak from 17.6 nm NCs and contains a clear resonance, matching the energy position and spectral shape of 5.3 nm NCs (donors), indicating that larger NCs are effectively excited by the energy funneling from the small NCs.

### Coherent NC–NC Coupling and Superfluorescence

**Figure 6:**
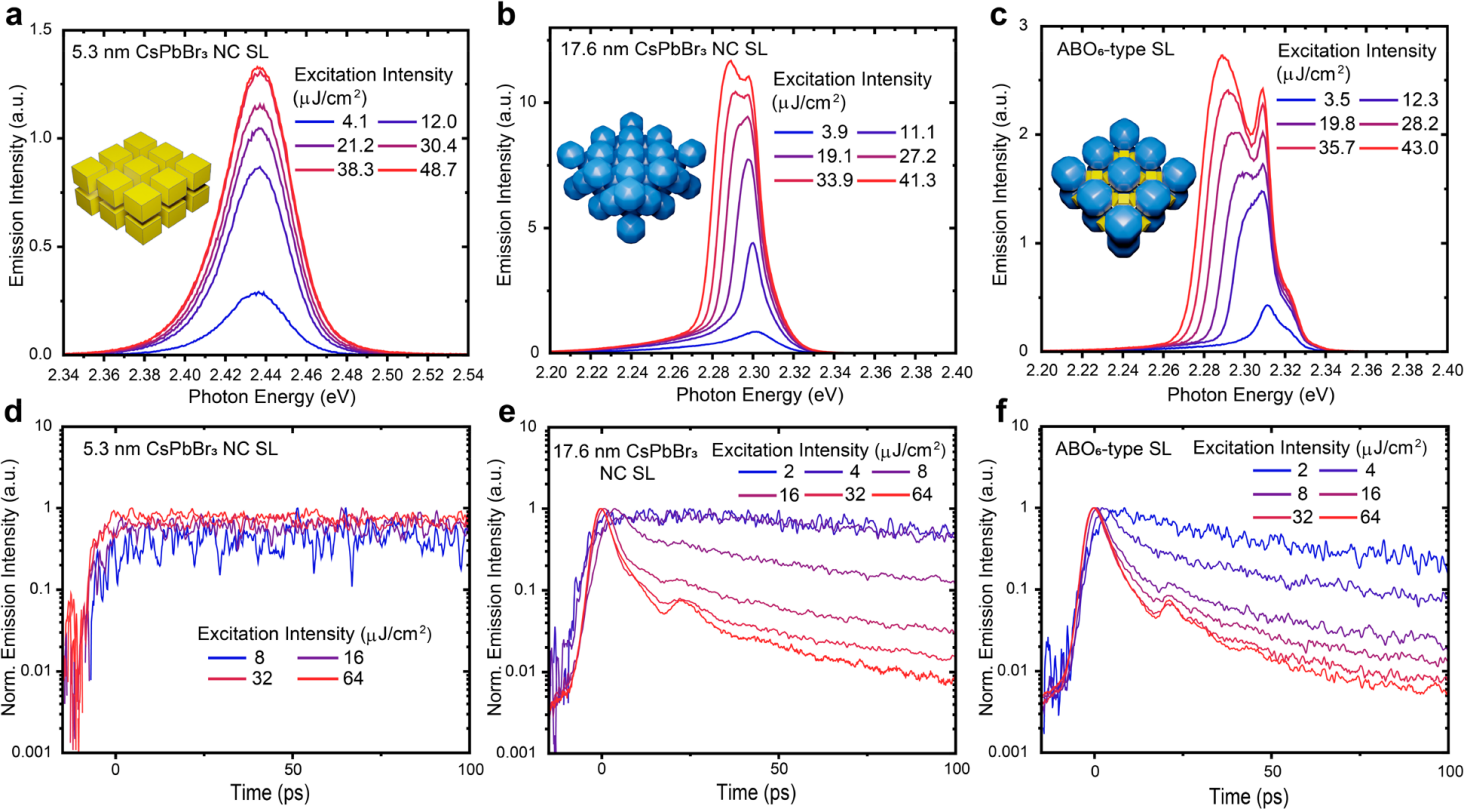
Time-resolved PL spectroscopy of reference single-component samples and binary ABO_6_-type SL in a high excitation regime at a cryogenic temperature (6 K). (a) PL spectra for 5.3 nm CsPbBr_3_ NC SL under different excitation intensities demonstrating the unchanged PL spectrum shape. (d) Spectrally integrated time-resolved emission intensity traces for 5.3 nm CsPbBr_3_ NC SL at different excitation intensities, showing no radiative lifetime shortening. (b and c) PL spectra for 17.6 nm CsPbBr_3_ NC SL and ABO_6_-type SL, respectively, revealing the appearance of a red-shifted emission band at higher excitation intensities. (e and f) Spectrally integrated time-resolved emission intensity traces of the red peak at different excitation intensities for 17.6 nm CsPbBr_3_ NC SL and ABO_6_-type SL, respectively, showing SF features: the shortening of the radiative lifetime and Burnham–Chiao ringing behavior.

Recently, single-component and binary SLs have emerged as a platform for exploring coherent NC–NC coupling. Perovskite NC SLs have broad structural and compositional engineerability, which has been exploited toward the development of more efficient superradiant SLs; SF has been indeed found to depend strongly on the perovskite NC density in the SLs.^29^ We probed the occurrence of SF in all-perovskite NC SLs by time-resolved PL spectroscopy. In a high excitation regime, while the reference 5.3 nm NCs do not show any changes in the PL spectral shape (Figure 6a) in line with previous observations,^27^ the reference 17.6 nm NCs and the ABO_6_-type SL exhibit clear signature for the occurrence of collective emission (Figure 6b,c). The time-resolved PL spectra from the reference 17.6 nm NCs and the ABO_6_-type SL exhibit a shortening of the 1/e radiative lifetime down to 5 ps and a ringing behavior in the time domain, typical features of SF (Figure 6e,f). Typical excitation fluence for the occurrence of SF is around 10 μJ/cm^2^ for the all-perovskite NC SLs, which is rather lower than for previously explored SLs. This is probably due to the much stronger oscillator strength of excitons in relatively large NCs^20^ and the lower inhomogeneous broadening for the exciton energy in larger NCs. The results indicate that the interactions among large NCs in the binary SL are sufficiently strong to support the emergence of SF, as the distance between 17.6 nm NCs in the binary SLs (ca. 21 nm) is comparable to the interparticle distance in the single-component 17.6 nm NC film.

### Exciton Diffusion Measurements in Mesoscopic Ordered SLs

**Figure 7:**
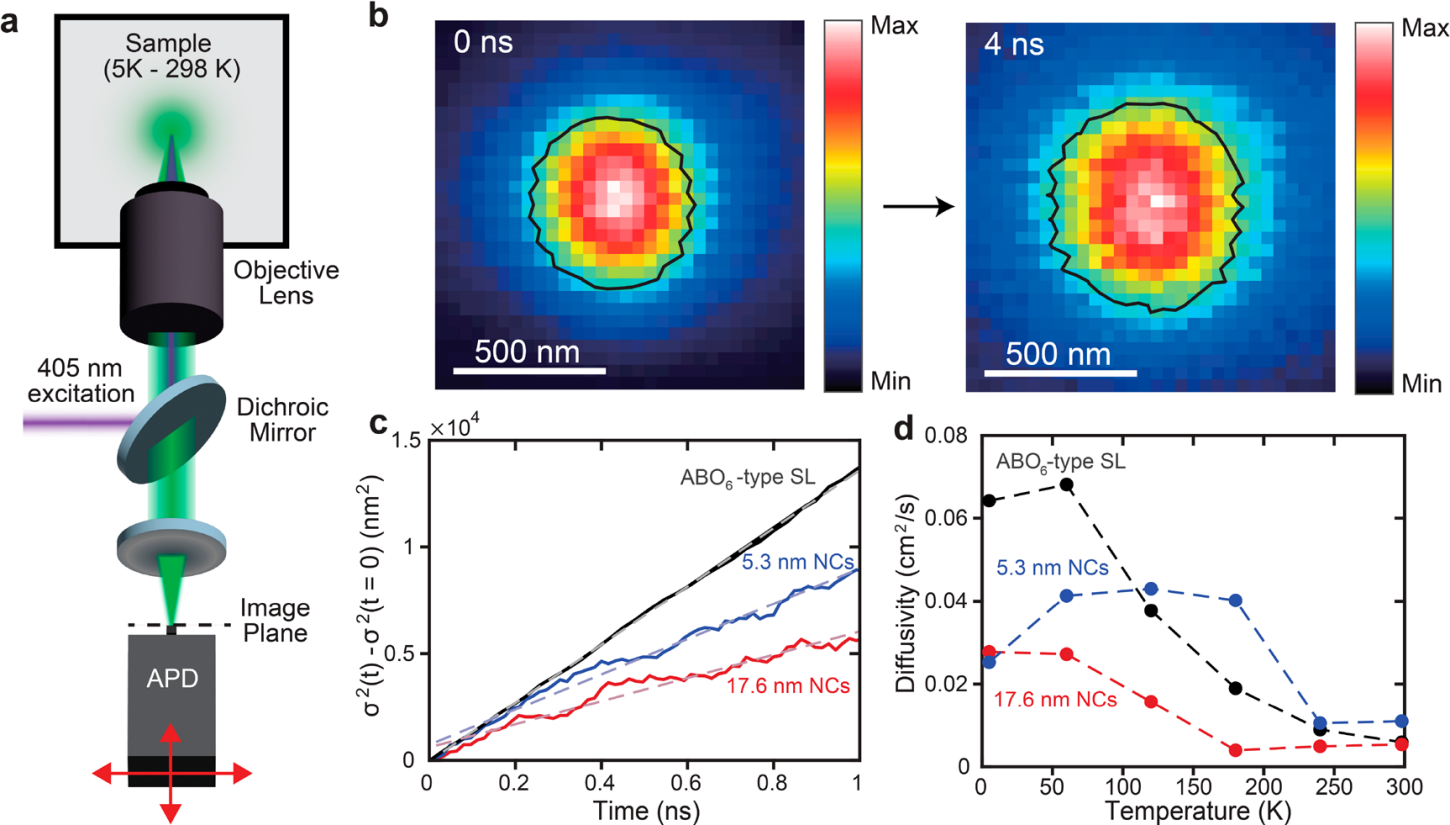
Probing exciton diffusion in all-perovskite multicomponent NC SLs. (a) Schematic of the PL microscope used for diffusion imaging. (b) PL intensity maps (normalized) from the binary SL at 5 K at two different delays after the laser pulse, showing the broadening of the spatial profile of the excitons. The black outlines denote the contours of the exciton profile at half of the maximum intensity. (c) Change in variance of PL profiles at 60 K for the ABO_6_-type SL and the reference samples of 17.6 nm NCs and 5.3 nm NCs. (d) Exciton diffusivity of the three samples as a function of temperature from 5 to 298 K.

The occurrence of strong excitonic interactions between small and large NCs in an ordered SL can also affect the diffusion of excitonic energy over longer distances. To probe the spatiotemporal dynamics of excitons, we employed transient PL microscopy (Figure 7a) to image exciton diffusion within NC assemblies at temperatures ranging from 5 to 298 K.

In this experiment, the spatial profile of excitonic emission is monitored on a picosecond-to-nanosecond time scale, enabling the measurement of the collective diffusivity of the exciton population (Figure 7b). To determine the impact of the SL structure on excitonic transport, we compared the exciton diffusivity in the binary ABO_6_-type SL containing both 17.6 and 5.3 nm NCs to the exciton diffusivity in reference samples containing only 17.6 nm NCs or only 5.3 nm NCs (Figure 7c). Because the time resolution of the transient PL microscopy experiment is ≥50 ps, far longer than the time scale for energy transfer from the 5.3 nm NCs to the 17.6 nm NCs, we expect spatiotemporal dynamics on these time scales to be dominated by interactions between the 17.6 nm NCs.

However, the measured exciton diffusivity within the binary SL was found to be larger than that in the 17.6 nm NC reference sample across all temperatures (Figure 7d). Furthermore, the difference in exciton diffusivity between the binary SL and the 17.6 nm NC assembly became especially pronounced at a lower sample temperature. At the lowest temperatures (≤60 K), the binary SL had a greater diffusivity than either the 5.3 nm NC sample or the 17.6 nm NC reference sample alone, suggesting that excitonic transfer through the 5.3 nm NCs is not the primary cause for the enhanced exciton diffusivity in the binary SL.

The differences in exciton diffusivity that emerge between the samples at lower temperatures could reflect the impact of excitonic coherence on the energy transport in these materials. At low temperatures, the increased amount of coherent coupling in the binary SL and the 17.6 nm NC assembly, mediated by the higher oscillator strength in large NCs,^20,79^ would be expected to increase the exciton diffusivity.^80^ This may explain why these samples exhibited the highest exciton diffusivities at low temperatures, while the 5.3 nm NC assembly, which did not exhibit key signs of excitonic coherence and superfluorescence, had a decrease in exciton diffusivity at lower temperatures.

As a final remark, efficient and directional streaming of energy was demonstrated early on in LHP NCs by blending NCs of different sizes.^81^ In the binary SLs, the high structural order in the positions of the NC donor and acceptor allows for such energy channeling to be spatially highly uniform, on top of being efficient and directional. This is demonstrated by hyperspectral PL mapping experiments performed on binary SL and disordered film comprising 5.3 and 17.6 nm NCs. The experimental setup along with PL spectra, PL peak wavelength and intensity maps obtained from the two samples at cryogenic temperatures are displayed in Figure S14. It is evident that the ordered arrangement of the two NCs within the superstructure yields a much more uniform spread of energy, resulting in a uniform distribution of PL intensity and wavelength, largely dominated by the energy acceptor, i.e., the exciton emission of the 17.6 nm NC component. On the other hand, the disordered sample exhibits inhomogeneous emission and phase-separated areas containing mainly only the small or only large NC emission.

## Conclusions

In summary, this study introduces the LHP NC-only multicomponent SLs. To enhance the SL formability and achieve packing densities exceeding those of single-component SLs, we employed nanocubes as the smaller SL component and rhombicuboctahedral (quasi-spherical) NCs as the larger counterpart. Depending on the selected particle number ratio, we obtained ABO_6_-type and NaCl-type SLs in a pure phase form using 5.3 and 17.6 nm CsPbBr_3_ NCs. The increase in the effective size ratio to γ_eff_ ≈ 0.5 by employing larger 8.0 nm CsPbBr_3_ nanocubes resulted in the formation of a perovskite-type ABO_3_ structure, while a NaCl structure formed at a lower small-to-large NC number ratio. The described SLs exhibit a high degree of positional and orientational ordering of the constituent NCs compared with previously reported cube-sphere NC SLs. For ABO_6_-type SL, a model system containing two different types of luminescent NCs, we explored NC coupling within the SL. Time-resolved measurements at both room and cryogenic temperatures provide evidence for efficient energy transfer from the strongly confined 5.3 nm CsPbBr_3_ NCs to the less confined 17.6 nm CsPbBr_3_ NCs. In a strong excitation regime, the ABO_6_-type SL exhibits key signatures of SF, corroborating the occurrence of collective emission behavior. The exciton dynamics was probed with transient PL microscopy and revealed higher excitonic diffusivity values in the binary SLs compared to single-component 17.6 and 5.3 nm NC reference samples (at temperatures below 60 K), presumably as a result of a stronger exciton coherence and a high degree of structural order. These findings will thus guide the further engineering of multicomponent SLs with tailored excitonic properties.

## Methods

### Safety Statement

No unexpected or unusually high safety hazards were encountered.

### Preparation of Binary CsPbBr_3_–CsPbBr_3_ SL

Binary SLs were prepared by means of a drying-mediated approach, whereby the NC solution was slowly evaporated over a tilted support. TEM grids (F/C-coated, Ted Pella, with the Formvar layer removed by immersing the grid in toluene for 10 s) or SiN membranes (Agar Scientific, Norcada) were employed as the substrates. A coassembly was carried out by placing the NC mixture in toluene (30–35 μL) into a 2 mL vial with solid support inside. The vial was then positioned tilted in the vacuum chamber (pressure ∼0.5 bar, room temperature), where it was left until all solvent evaporated. The mixtures contained overall NC concentrations in the 0.8–1 μM range. For binary ABO_6_-type SL, 5.3 nm CsPbBr_3_ NCs (9.6 μM, 3 μL), 17.6 nm CsPbBr_3_ NCs (0.9 μM, 4 μL), and anhydrous toluene (25 μL) were used. For NaCl-type SL, a lower small-to-large NC number ratio was employed: 5.3 nm CsPbBr_3_ NCs (9.6 μM, 2 μL), 17.6 nm CsPbBr_3_ NCs (1.3 μM, 6.2 μL), and anhydrous toluene (25 μL). For binary ABO_3_-type SL, larger 8.0 nm CsPbBr_3_ NCs (10.1 μM, 3 μL) were utilized together with 17.6 nm CsPbBr_3_ NCs (1.1 μM, 5 μL), and anhydrous toluene (25 μL).

### Microscopy Characterization

TEM and STEM images, as well as WAED and SAED patterns, were collected with a JEOL JEM 2200FS electron microscope operating at an accelerating voltage of 200 kV. Image analysis was performed using ImageJ, and ED patterns were compared with the simulated ones in Crystal Maker and Single Crystal Software. HAADF-STEM images were recorded with an FEI Titan Themis electron microscope operating at 300 kV. Template matching and image averaging was done by using MacTempas software (Total Resolution LLC). SEM images were collected with an FEI Helios 660 microscope in immersion mode at 6 kV. Electron tomography was carried out in HAADF-STEM mode. HAADF-STEM images at different tilt angles were manually recorded with the aid of a motorized dual-axis tomography holder using an FEI Titan Themis microscope operated at 300 kV. A small beam semiconvergence angle of 2.5 mrad was used, which resulted in a depth of field of 315 nm. HAADF-STEM images (2048 × 2048 pixels, 2.28 Å pixel size, 2.01 s frame time) were recorded at 2° tilt intervals over a range from −63° to +65° at an electron probe current of <10 pA. Thus, for the whole tilt series the electron dose resulting from image acquisition only was calculated to be 301 electrons/Å^2^. Since at each tilt position it was necessary to correct the position of the region of interest, we estimate that the total electron dose was 4 to 5 times higher. Image alignment was performed using the band-pass filter routine of the Digital Micrograph software, and the 3D volume reconstruction was carried out by means of the total variation minimization (TVM) reconstruction technique implemented in TomoJ, a plug-in for the ImageJ software. The 3D volume rendering and orthoslices were generated with the Avizo 3D visualization program.

### GISAXS and GIWAXS Characterization

Binary all-perovskite NC SLs were probed on 50 nm thick SiN membranes, NT050A, supplied by Norcada. Grazing incidence X-ray diffraction (GIXRD) data were collected at the Swiss-Norwegian BM01 beamline at the European Synchrotron Radiation Facility with 13 keV X-ray radiation and a Pilatus 2 M Silicon detector set at ca. 640 mm from the center of the sample. The standard NIST LaB_6_ powder was used to finely determine the wavelength and to calibrate the detector. Calibrated data were analyzed by the GIDVIS software.^82^

### Pump–Probe Spectroscopy

Pump–probe differential transmission spectroscopy was carried out using a Ti:sapphire-based ultrafast amplifier generating 100 fs pulses centered at 800 nm at a 1 kHz repetition rate. The output beam from the amplifier was transmitted through a half-waveplate and a thin film polarizer, splitting the beam into pump and probe pulses. The half waveplate allowed precise control of the energy directed into the probe optical path required for generating stable white light when focused onto a 2 mm thin sapphire plate. The collimation and focusing of the white light probe beam onto the sample were achieved by using parabolic mirrors to minimize dispersion effects. The pump beam followed an optical path that included a motorized translation stage with 0.1 μm resolution, introducing a controlled delay between the arrival of the excitation pulse and the probe pulse with subfemtosecond resolution. A second half-waveplate and thin film polarizer pair was utilized before a second-harmonic generation crystal (BBO) in the pump beam optical path, thereby controlling the energy of the 400 nm incident on the sample. Both the pump and probe beams were directed on the sample, which was placed in a cryostat for tuning the sample temperature in the 77 to 300 K range. Careful alignment of the pump beam through the translation stage ensured that the probe beam of 100 μm in diameter was always within the larger spot diameter of 1.5 mm of the pump beam. The measurements were carried out using a typical pump–probe optical setup in a noncollinear configuration with the pump arm incorporating an optical chopper synchronized at half the frequency of the ultrafast amplifier. The white light probe beam following its transmission through the sample was directed into a fiber-optic coupled spectrometer with 0.5 nm spectral resolution equipped with a fast CCD array, thereby providing measurements over a broad range of wavelengths. The synchronized optical chopper allowed transmission measurements of two consecutive pulses with and without sample excitation, thus achieving a signal-to-noise ratio of 104 over an interval of a few seconds.

### Time-Resolved PL

The sample was mounted in a helium exchange-gas cryostat at 6 K. For the SF experiments, a frequency-doubled regenerative amplifier seeded with a mode-locked Ti:sapphire laser with a pulse duration of 100–200 fs and a repetition rate of 1 kHz at 3.1 eV photon energy was used as an excitation source. For the weak fluence energy transfer measurements, the same system was used without the regenerative amplifier, resulting in an 80 MHz pulse repetition rate. The excitation light was passed through short-pass filters (442 nm cutoff wavelength). For both excitation and detection, we used the same focusing lens with a 100 mm focal length, resulting in an excitation spot radius of about 60 μm. The recorded PL was long-pass filtered (480 nm cutoff wavelength) and then dispersed by a grating with 150 lines per mm in a 0.3 m long monochromator and detected with a streak camera with a nominal time resolution of 2 ps and instrument response function fwhm of 4 ps. The time-integrated PL spectra were recorded by a 0.5 m long spectrograph with a grating with 300 lines per millimeter and a nitrogen-cooled CCD camera.

### PL Mapping

Two-dimensional hyperspectral PL maps were acquired on a custom-made confocal PL system equipped with a high precision x-y-z motorized stage enabling the collection of PL spectra every 150 nm in the lateral direction and 250 nm in the vertical direction. Excitation and collection were performed via the same long working distance 50× (NA = 0.55) objective, focusing the excitation laser down to a diffraction-limited spot of approximately 1 μm in diameter. A 405 nm continuous wave laser diode coupled to a single mode fiber was used as the excitation source, with the emission spectra detected via a combination of a 0.75 m Acton750i Princeton spectrometer and a 1024 × 256 pixels PIXIS charge-coupled device (CCD) camera. The samples were kept in a closed-loop helium cryostat, allowing continuous temperature variation from 10 to 300 K.

### PLE/PL Measurements

Photoluminescence excitation (PLE) measurements were recorded on a FluoroLog FL3 Horiba Jobin Yvon spectrofluorometer using a 450 W ozone-free Xenon Lamp, filtered via a double grating spectrometer as the excitation source. The excitation source was coupled onto a 50 μm multimode fiber, with the excitation and collection performed under the confocal PL setup described above.

### Single-QD Spectroscopy

A custom-built μ-PL set-up was used. The samples were mounted on xyz nano-positioning stages inside an evacuated liquid-helium closed-loop cryostat (Montana Instruments) and cooled down to a targeted temperature of 4 K. Single NCs were excited using a fiber-coupled excitation laser, which is focused (1/e^2^ diameter = 2.4 μm) on the sample by a microscope objective (NA = 0.8, 100×). The emitted light was collected by the same objective and passed through a dichroic mirror (long-pass, cut-on wavelength 450 nm long-pass filter). A monochromator coupled to an EMCCD (Princeton Instruments, 0.5-m, 1 s binning time) was used for spectra measurements. A single APD (MPD, time resolution of 50 ps) mounted after the monochromator, which accepts photons only from the exciton photoluminescence, was used to measure TRPL traces. A HBT set-up with a 50/50 beam splitter, two APDs and a TCSPC Module (PicoQuant) was used for second-order correlation (g^(2)^(τ)) measurements.

### Exciton Diffusion Imaging

SL samples were mounted on a piezo stage (attocube, ANC350) and kept under a vacuum inside a closed-cycle liquid helium cryostat (Montana Instruments Cryostation s100) (Figure 7a). For low-temperature experiments, the cryostat was used in conjunction with a temperature controller (Lakeshore Model 335) to maintain the temperature. To collect transient PL spectra, first, a 405 nm pulsed laser (PicoQuant LDH–P-C-405M, driven by a PDL 800-D, pulse width <100 ps) at 10 MHz and a fluence of 0.094 μJ/cm^2^ was focused onto a near diffraction-limited spot on the sample using an objective lens (Zeiss EC Epiplan-Neofluar 100×/0.85 NA). Photoluminescence was collected by the same objective, filtered with a dichroic (Semrock Di02-R405) and a long-pass filter (Thorlabs FGL435M), magnified with a telescope (Thorlabs AC254-030-A and AC254-125A), and focused onto an avalanche photodetector (APD, Micro Photon Devices, ∼50 ps time resolution). The active area of the APD (50 μm × 50 μm) was raster scanned across the PL image using two motors (Thorlabs ZFS25B). The spatial distribution of the exciton profile over time was then reconstructed by combining the PL decay curves from each point along the scan.

To determine the exciton diffusivity, first, the spatial profile at each time was fit to a Gaussian profile of the form
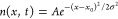
where *A* is the profile height, *x*_0_ is the profile center, and σ^2^ is the profile variance. The diffusivity of a Gaussian profile of excitons is given by the rate of increase in the spatial variance over time:
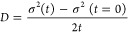


To determine the diffusivity, a line was fit to the changes in variance σ^2^ within the first few nanoseconds following laser excitation.

## Supporting Information Available

Supplementary details on NC building blocks (detailed synthesis, EM, and optical characterizations), additional NaCl and ABO_3_ structures, space-filling analysis, image averaging of binary SL, EM imaging of segregated cubic-shaped NCs, GIWAXS and GISAXS data for single-component and binary SLs, time-resolved PL spectroscopy for reference samples and binary SL, PL and PLE spectra for binary SL at cryogenic temperature, and hyperspectral PL mapping (PDF)
Video S1: tomography reconstruction of ABO_6_-type SL domain (AVI)

## Supplementary Material

nn3c13062_si_001.pdf

nn3c13062_si_002.avi
