# Supplementary material for: All-Perovskite Multicomponent Nanocrystal Superlattices
Source: ACS Nano. 2024 Mar 6;18(11):8423–36. doi: 10.1021/acsnano.3c13062 (PMC10958606; doi:10.1021/acsnano.3c13062)
Supplement: Supplementary file 1 — nn3c13062_si_001.pdf [file nn3c13062_si_001.pdf]

Supporting Information

All-Perovskite Multicomponent Nanocrystal Superlattices

Taras V. Sekh,[1,2] Ihor Cherniukh,[1,2] Etsuki Kobiyama,[3] Thomas J. Sheehan,[4] Andreas Manoli,[5] Chenglian Zhu,[1,2] Modestos Athanasiou,[5] Marios Sergides,[6] Oleksandra Ortikova,[7] Marta D. Rossell,[7] Federica Bertolotti,[8] Antonietta Guagliardi,[9] Norberto Masciocchi,[8] Rolf Erni,[7] Andreas Othonos,[6] Grigorios Itskos,[5] William A. Tisdale,[4] Thilo Stöferle,[3] Gabriele Rainò,[1,2] Maryna I. Bodnarchuk,[1,2*] Maksym V. Kovalenko[1,2*]

[1] Institute of Inorganic Chemistry, Department of Chemistry and Applied Biosciences, ETH Zürich, 8093 Zürich, Switzerland

[2] Laboratory for Thin Films and Photovoltaics, Empa, Swiss Federal Laboratories for Materials Science and Technology, 8600 Dübendorf, Switzerland

[3] IBM Research Europe - Zurich, Rüschlikon CH-8803, Switzerland

[4] Department of Chemical Engineering, Massachusetts Institute of Technology, Cambridge, Massachusetts 02139, United States

[5] Experimental Condensed Matter Physics Laboratory, Department of Physics, University of Cyprus, 1678 Nicosia, Cyprus

[6] Laboratory of Ultrafast Science, Department of Physics, University of Cyprus, Nicosia 1678, Cyprus

[7] Electron Microscopy Center, Empa−Swiss Federal Laboratories for Materials Science and Technology, CH-8600 Dübendorf, Switzerland

[8] Department of Science and High Technology and To.Sca.Lab, University of Insubria, via Valleggio 11, 22100 Como, Italy

[9] Istituto di Cristallografia and To.Sca.Lab, Consiglio Nazionale delle Ricerche, via Valleggio 11, 22100 Como, Italy

[*]corresponding authors: mvkovalenko@ethz.ch, maryna.bodnarchuk@empa.ch

NC building blocks for SL

Chemicals

Cs_2CO_3 (99.9%, Aldrich), $PbBr_2$ (99.999%, Aldrich), PbO (99.999%, Aldrich), $ZnBr_2$ (99.9%, Alfa Aesar), phenacyl bromide (98%, Aldrich), didodecyldimethylammonium bromide (98%, Aldrich), oleic acid (OA, 90%, Aldrich), oleylamine (OLA, min. 95%, Strem), ethyl acetate (HPLC Plus, 99.9%, Aldrich), hexane (anhydrous, 95%, Aldrich), mesitylene (99%, Acros Organics), 1-octadecene (ODE, for synthesis, Aldrich), toluene (anhydrous, 99.8%, Aldrich).

Preparation of 0.12M Cs-OA stock solution, adapted from Ref.[1]
195.4 mg (0.6 mmol) of Cs_2CO_3 was loaded in a 25 ml round-bottom flask along with 1 ml OA (vacuum-dried at 100 °C) and 9 ml ODE (distilled). The reaction mixture was purged with N_2 for 1 h at 120 °C and 10 min at 150 °C. The solution was then cooled to room temperature and stored in the glovebox.

Preparation of 0.15M Cs-OA stock solution, adapted from Ref.[1] 200.0 mg (0.614 mmol) of Cs_2CO_3 was loaded in a 25 ml round-bottom flask along with 0.6 ml OA (vacuum-dried at 100 °C) and 7.5 ml ODE (distilled). The reaction mixture was degassed for 20 min at 100 °C and then purged with N_2 at 120 °C until all the Cs_2CO_3 reacted with OA. The solution was then cooled to room temperature and stored in the glovebox.

Synthesis of 5.3 nm $CsPbBr_3$ NCs, adapted from Ref. [2, 3] In a 25 ml three-neck flask, 75 mg of $PbBr_2$ (0.2 mmol), and 180 mg of $ZnBr_2$ (0.8 mmol) were suspended in 5 mL of mesitylene (dried) and degassed five times at room temperature. The suspension was quickly heated under an N_2 atmosphere, and when the temperature reached 120 °C, 2 ml of OA (vacuum-dried at 100 °C) and 2 ml of OLA (distilled) were injected. Then, at 146 °C, 0.4 ml of a 0.15M Cs-OA solution in ODE (preheated to about 100 °C) was swiftly injected. The reaction mixture was immediately cooled to room temperature with an ice-water bath. The crude solution was centrifuged at 12100 rpm (20130 rcf) for 3 min, the precipitate was discarded, and the supernatant was destabilized with 27.5 ml of ethyl acetate. The solution was centrifuged again at 12100 rpm (20130 rcf) for 5 min, the supernatant was discarded, and the precipitate was redispersed in 1.8 ml of anhydrous toluene. Subsequently, the OLA/OA ligands were exchanged by treatment with DDAB. For that, 90 µl of 0.01M DDAB solution in toluene was added to the NC dispersion. The solution was then stirred for 2 h, followed by centrifugation at 12100 rpm (20130 rcf) for 6 min and discarding the precipitate.

Synthesis of 8.0 nm $CsPbBr_3$ NCs, adapted from Ref.[1, 4] In a 25 mL three-neck flask, 55 mg of $PbBr_2$ (0.15 mmol) was degassed three times, suspended in 5 ml of ODE (distilled), and degassed three times again at room temperature. The suspension was quickly heated under an N_2 atmosphere, and when the temperature reached 120 °C, 0.5 ml of OA (vacuum-dried at 100 °C) and 0.5 ml of OLA (distilled) were injected. Then, at 180 °C, 0.6 ml of 0.15M Cs-OA solution in ODE (preheated to about 100 °C) was swiftly injected. The reaction mixture was cooled immediately to room

temperature with an ice-water bath. The crude solution was centrifuged at 12100 rpm (20130 rcf) for 5 min, the supernatant was discarded, and the precipitate was dispersed in 0.3 ml of anhydrous hexane. The solution was centrifuged again at 10000 rpm (13780 rcf) for 3 min, and the precipitate was discarded. Then, OLA/OA ligands were exchanged by DDAB treatment. For that, 0.3 ml of anhydrous hexane, 0.6 ml of anhydrous toluene, and 0.14 ml of 0.05M DDAB solution in toluene were added to the supernatant. The solution was then stirred for hour, followed by destabilization with 1.8 ml of ethyl acetate, centrifugation at 12100 rpm (20130 rcf) for 3 min, and redispersion in 0.6 mL of anhydrous toluene.

Synthesis of 14-18 nm $CsPbBr_3$ NCs, adapted from Ref.[5] In a 25 mL three-neck flack, 44.6 mg of PbO (0.2 mmol), 119.4 mg of phenacyl bromide (0.6 mmol), 1 ml of OA (vacuum-dried at 100 °C) were loaded and suspended in 5 ml of ODE. The temperature was increased to 220 °C, and 0.6 ml of oleylamine (distilled) was injected into the reaction mixture. The solution initially became red, then gradually turned orange and yellow in ~10 min. The temperature was decreased to 210 °C and 0.5 ml of 0.12M Cs-OA solution in ODE (preheated to about 100 °C) was swiftly injected into the bright yellow solution. The solution was annealed for 1 h at 210 °C and then cooled down to room temperature with an ice-water bath. The crude solution was centrifuged at 3000 rpm (1240 rcf) for 5 min, the dark-brown supernatant was discarded, and the precipitate was redispersed in 2 ml of toluene. The solution was centrifuged a second time at 3000 rpm (1240 rcf) for 3 min, and the precipitate was discarded. The OLA-OA ligands were exchanged by DDAB/$PbBr_2$ treatment. DDAB+$PbBr_2$ stock solution was prepared by dissolving 36.7 mg (0.1 mmol) $PbBr_2$ and 92 mg (0.2 mmol) DDAB in 3 mL of anhydrous toluene. 60 µl of DDAB/$PbBr_2$ solution was added to the NC dispersion. The solution was then stirred for 1 h, followed by destabilization with 1.2 ml of ethyl acetate, centrifugation at 12100 rpm (20130 rcf) for 2 min, and redispersion in 0.4 ml of toluene. Smaller 14.2 nm $CsPbBr_3$ NCs were obtained by annealing at a lower temperature (205 °C).

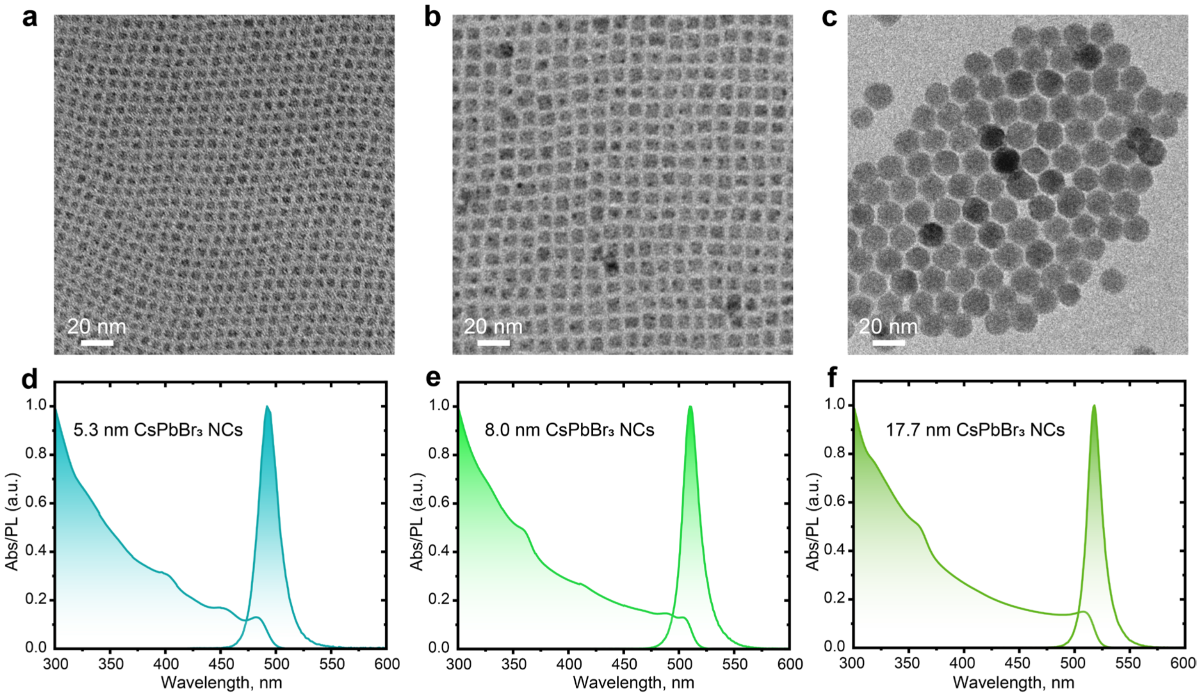

Figure S1. **NC building blocks for SL.** (a) TEM image of 5.3 nm $CsPbBr_3$ NC monolayer with (d) the corresponding absorption and PL spectra of the NC solution in toluene. (b) TEM image of 8.0 nm $CsPbBr_3$ NC monolayer with (e) the corresponding absorption and PL spectra of the NC solution in toluene. (c) TEM image of 17.7 nm $CsPbBr_3$ NC monolayer with (f) the corresponding absorption and PL spectra of the NC solution in toluene. As elaborated elsewhere,[3] 5.3 nm $CsPbBr_3$ NCs exhibit unusual rhombic packing with an obtuse angle of ~104°, while 8.0 nm NCs preserve typical square ordering.[1] The 26-faceted 17.7 nm $CsPbBr_3$ NCs post-treated with DDAB are packed in a hexagonal lattice.

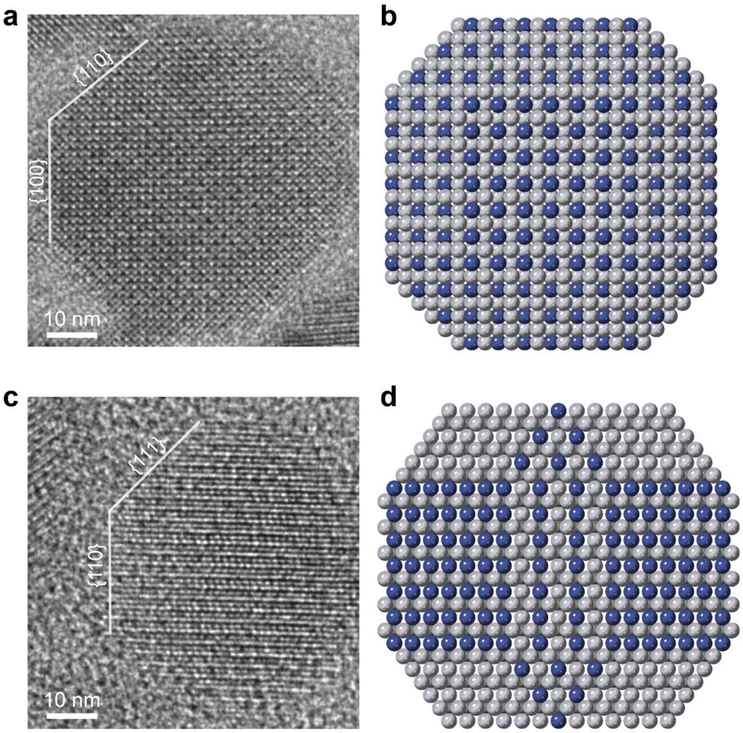

Figure S2. **Faceting of rhombicuboctahedral $CsPbBr_3$ NCs.** (a) HRTEM image of a NC viewed along $[100]_{NC}$ with (b) the corresponding structural model. (c) HRTEM image of a NC viewed along $[110]_{NC}$ with (d) the corresponding structural model. As evidenced by HRTEM images of NCs oriented along two crystallographic directions, the synthesized large-component $CsPbBr_3$ NCs exhibit a faceted rhombicuboctahedral shape.

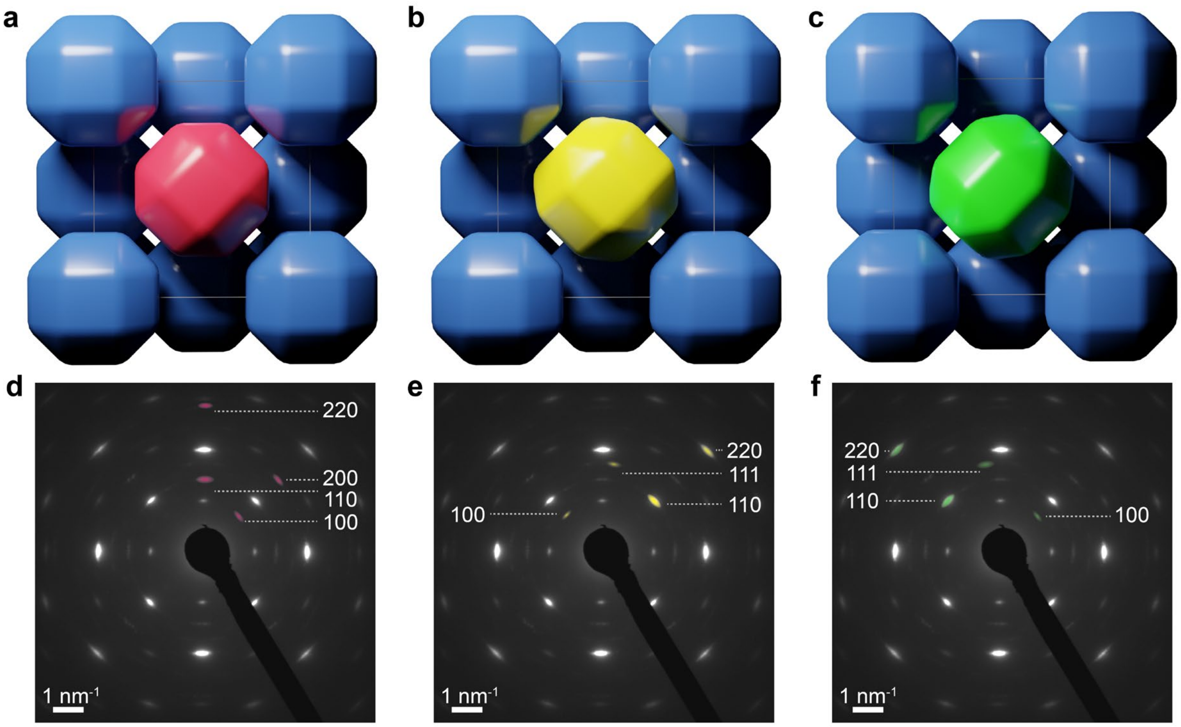

Figure S3. **Minor orientations of rhombicuboctahedral $CsPbBr_3$ NCs in a single-component SL.** (a) Structural model of the *fcc*-type unit cell with one NC rotated by 45° about the $[001]_{SL}$ zone-axis and (d) ED pattern with the corresponding reflections for this orientation (highlighted in magenta). (b) Structural model of the *fcc*-type unit cell with one NC, first, rotated by 45° about $[010]_{SL}$, then by 45° about the $[001]_{SL}$ zone-axis, and (e) ED pattern with the corresponding reflections for this orientation (highlighted in yellow). (c) Structural model of the *fcc*-type unit cell with one NC, first, rotated by 45° about $[010]_{SL}$, then by -45° about the $[001]_{SL}$ zone-axis, and (e) ED pattern with the corresponding reflections for this orientation (highlighted in green). The orientation in (a) is easily identifiable by the presence of a characteristic 110 reflection at 45° from the same reflection of the major orientation. The orientations in (b) and (c) are detected by the presence of 111 reflections, which are absent for the major orientation.

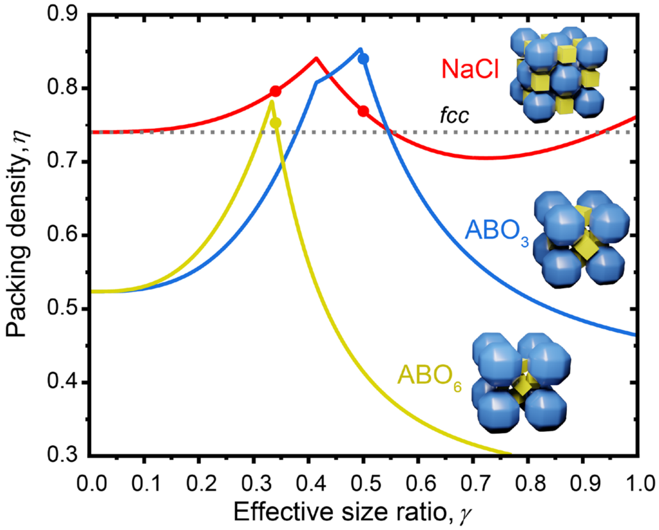

Figure S4. **Space-filling curves of NaCl- (red), ABO_3- (blue), and ABO_6- (yellow) type binary SLs** assembled from cubic and spherical NCs, with experimentally observed points depicted as circles. The packing density for *fcc*-packed spherical NCs is given as a grey dashed line. The space-filling curves for ABO_3 and ABO_6-type SLs were plotted within OTM, while the curve for NaCl-type was constructed using the hard-sphere model.[6] The packing density η is calculated as the volume fraction occupied by the NCs in the SL, leaving (1-η) as voids. Rhombicuboctahedral $CsPbBr_3$ NCs have a high tendency to behave like spherical NCs during self-assembly. Therefore, in the calculation of packing density for the $CsPbBr_3$-$CsPbBr_3$ SLs their shape was approximated as spherical. The packing densities for all experimentally chosen γ values exceed the packing density for the *fcc* lattice ($\eta = 0.74$), thus theoretically confirming their formability.

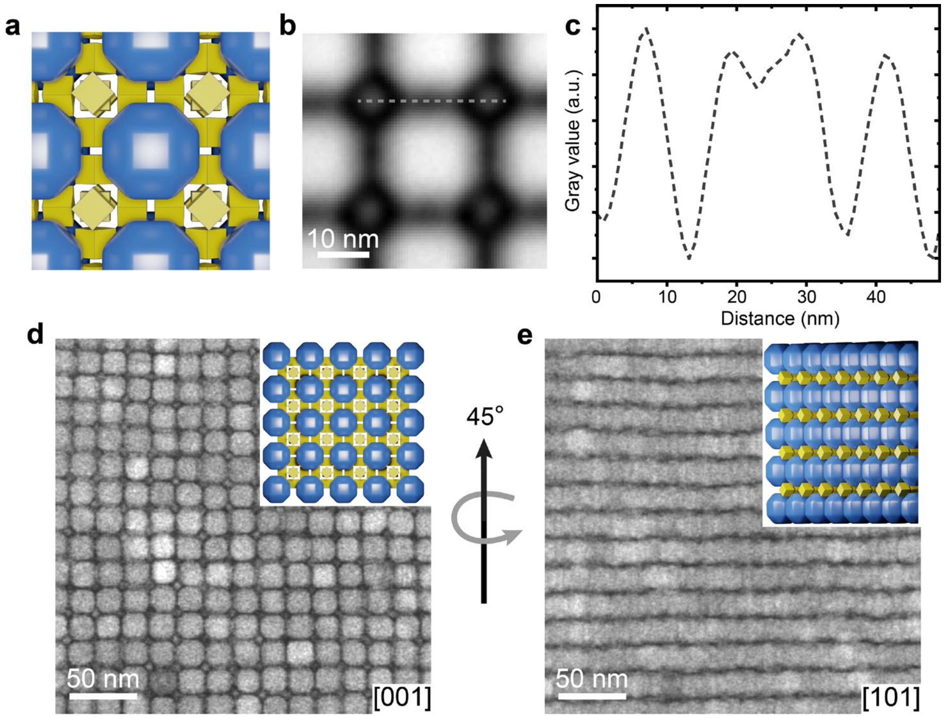

Figure S5. **Structural characterization of ABO_6-type SL.** (a) Structural model of a small area of the $[001]_{SL}$-oriented ABO_6-type SL domain. (b) Averaged HAADF-STEM image with (c) the corresponding line profile revealing the presence of two small O-site cubes positioned along the line between larger rhombicuboctahedral NCs. (d) HAADF-STEM image of $[001]_{SL}$-oriented domain showing the initial SL orientation with (inset) the corresponding structural model. (e) HAADF-STEM image of $[101]_{SL}$-oriented domain after tilting the substrate by 45° about $[010]_{SL}$ with (inset) the corresponding structural model.

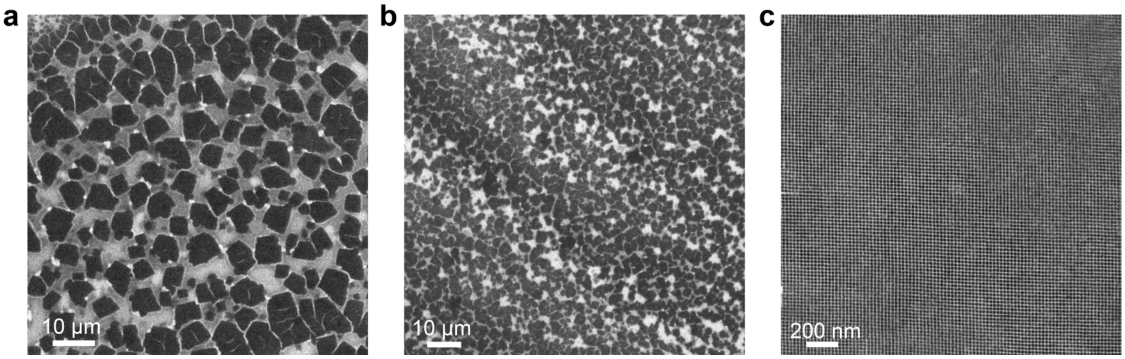

Figure S6. **ABO_6-type SL on SiN substrates.** (a and b) Low-magnification TEM images of ABO_6-type SL domains on Norcada SiN windows. (c) A close-up BF-STEM image of one of the SL domains. The images illustrate the extended SL domain coverage over SiN windows selected for subsequent optical measurements.

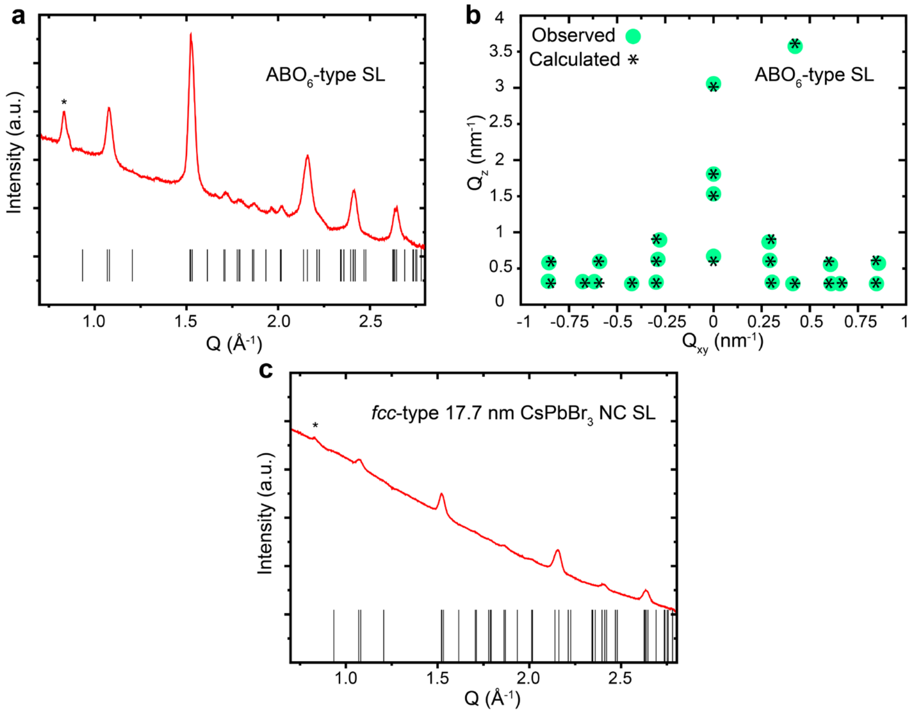

Figure S7. **GIWAXS and GISAXS data for single-component and ABO$_6$-type SLs.** Azimuthal-integrated 1D pattern in the WAXS region for (a) ABO$_6$-type SL and (c) single-component SL from 17.7 nm $CsPbBr_3$ NCs. Black ticks mark the theoretical *hkl* reflections for $CsPbBr_3$ with an orthorhombic crystal structure. $CsPbBr_3$ NCs in both SL types preserve the orthorhombic crystal structure, with the inclusion of the $CsPb_2Br_5$ phase possibly as the result of sample aging (peak at Q=0.85 Å, denoted as an asterisk). (b) The correlation between measured GISAXS reflections (green circles) and computed reflections (black asterisks) for ABO$_6$-type SL. The reflections are well-correlated, confirming the proposed cubic packing of larger NCs in the ABO$_6$-type SL, with unit cell edge of *ca.* 21 nm.

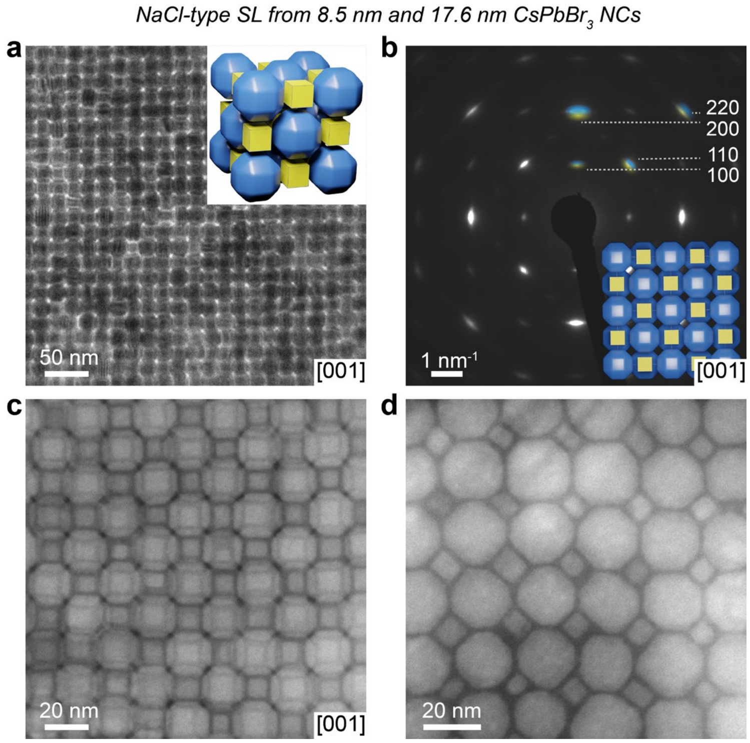

Figure S8. **NaCl-type $CsPbBr_3$-$CsPbBr_3$ SL comprising 8.5 nm and 17.6 nm $CsPbBr_3$ NCs.** (a) TEM image of $[001]_{SL}$-oriented NaCl-type SL domain with the unit cell shown in the inset. (b) Corresponding WAED pattern with the most intense reflections marked; superimposed reflections for 8.5 nm and 17.6 nm $CsPbBr_3$ NCs are shown in yellow and blue, respectively; indeed, the absence of extra peaks or Debye-Scherrer rings evidence the equioriented small and large NC SL components; (inset) structural model of a $[001]_{SL}$-oriented NaCl-type SL. (c) HAADF-STEM image of $[001]_{SL}$-oriented NaCl-type SL domain and (d) HAADF-STEM image of its monolayer confirming the formation of the binary SL.

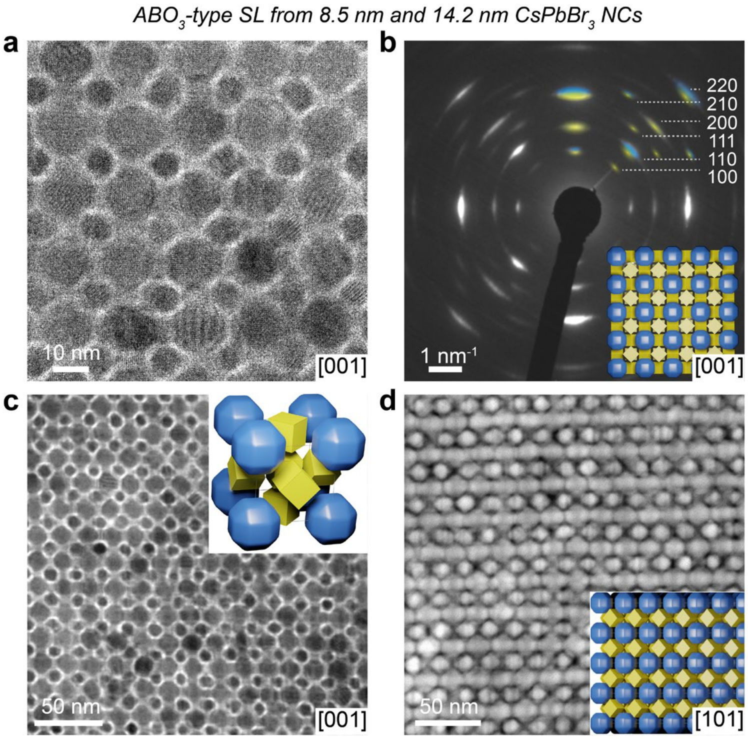

Figure S9. **ABO_3-type $CsPbBr_3$-$CsPbBr_3$ SL comprising 8.5 nm and 14.2 nm $CsPbBr_3$ NCs.** (a) TEM and (c) BF-STEM images of the $[001]_{SL}$-oriented ABO_3-type SL with (inset in c) the structural model of a unit cell. (b) The corresponding WAED pattern with the most intense reflections for 8.5 nm and 14.2 nm $CsPbBr_3$ NCs marked in yellow and blue, respectively; (inset) structural model of a $[001]_{SL}$-oriented ABO_3-type SL. (d) HAADF-STEM image of the ABO_3-type SL domain resembling $[101]_{SL}$ orientation with the corresponding structural model (inset). The high degree of orientational ordering is demonstrated in the WAED by the presence of sharp arcs corresponding to 110 and 200 reflections of A-site rhombicuboctahedra and B-site cubes, as well as by 111 reflections of O-site cubes.

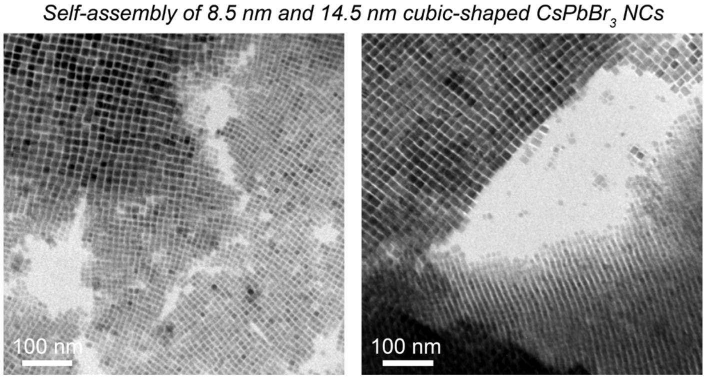

Figure S10. **Segregation of 8.5 nm and 14.5 nm $CsPbBr_3$ NCs**. TEM images showing the segregation of cubic-shaped 8.5 nm and 14.5 nm $CsPbBr_3$ NCs into single-component SLs during the co-assembly process.

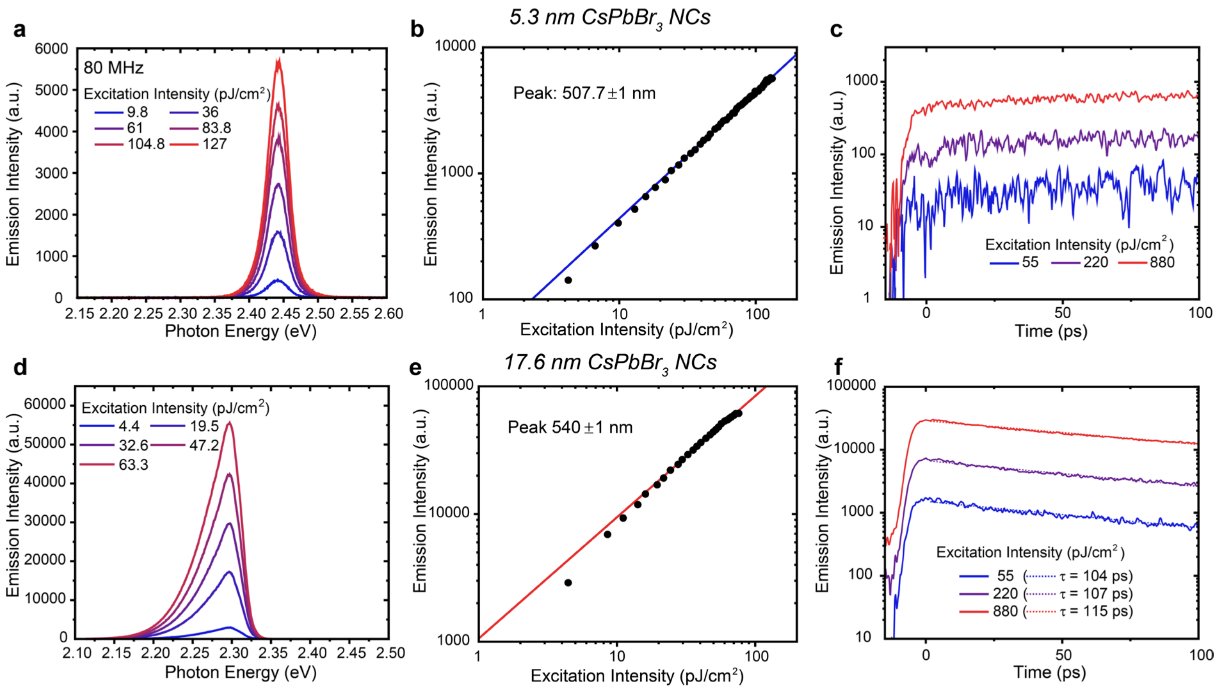

Figure S11. **Time-resolved PL spectroscopy of reference NC samples in a weak excitation regime at a cryogenic temperature (6 K).** (a and d) PL spectra for 5.3 nm and 17.6 nm $CsPbBr_3$ NCs, respectively, under different excitation intensities. (b and e) Fluence dependence of the PL peak intensity for 5.3 nm and 17.6 nm $CsPbBr_3$ NCs, where the solid lines denote power law fits with exponents of 1.00 and 0.95, respectively. (c and f) Spectrally integrated time-resolved emission intensity traces at different excitation intensities.

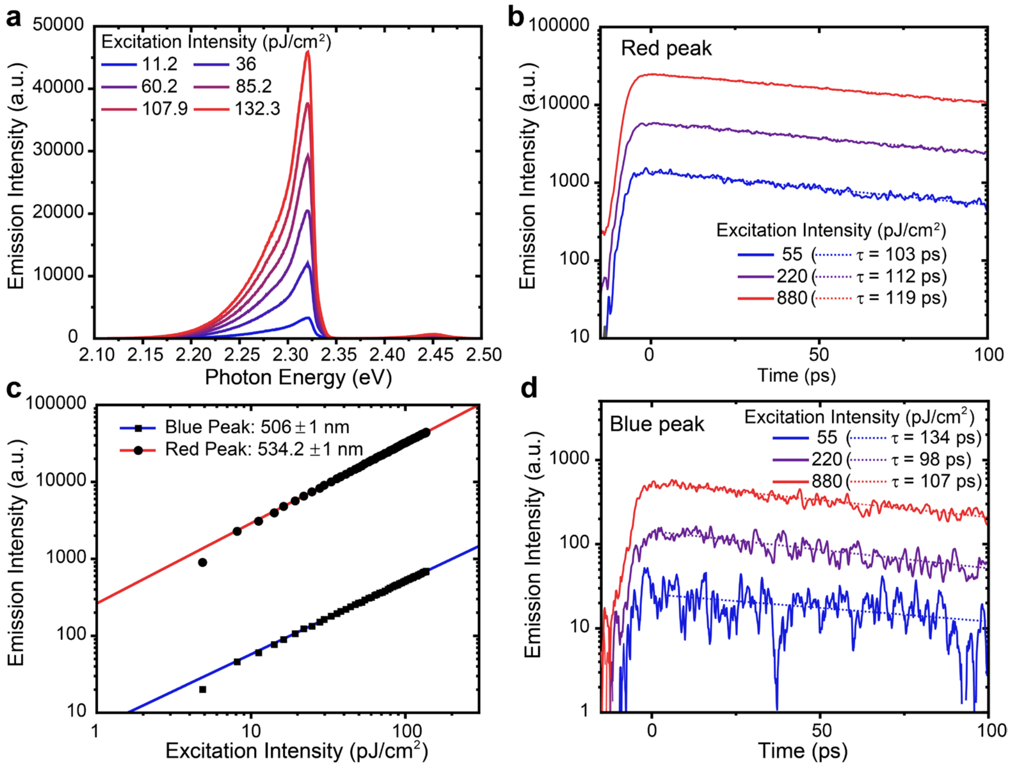

Figure S12. **Time-resolved PL spectroscopy of ABO_6-type SL in a weak excitation regime at a cryogenic temperature (6 K).** (a) PL spectra for ABO_6-type SL under different excitation intensities. (c) Fluence dependence of the PL peak intensity for red and blue peaks where the solid lines denote power law fits with exponents of 0.95 and 1.04, respectively. (b and d) Spectrally integrated time-resolved emission intensity traces at different excitation intensities for red and blue peaks, respectively. A drastic change in the 5.3 nm NCs (donor) lifetime in all-perovskite SLs compared to the reference sample (Figure S11c), attests to the occurrence of an efficient energy transfer also at cryogenic temperatures, in agreement with room temperature results obtained by pump-probe spectroscopy.

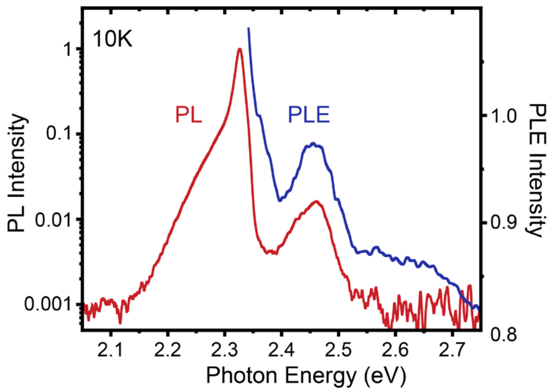

Figure S13. **PL (in logarithmic scale) and PLE spectra obtained at 10 K from ABO_6-type SL.** The PLE spectrum was obtained while monitoring the emission peak of the 17.6 nm NC component of the SL and contains a feature at ~2.45 eV matching the energy position and spectral shape of the 5.3 nm NC emission. The coincidence of the PL and PLE peaks confirm that the 17.6 nm NCs (energy acceptors) are effectively excited *via* energy funneling from the 5.3 nm NCs (energy donors).

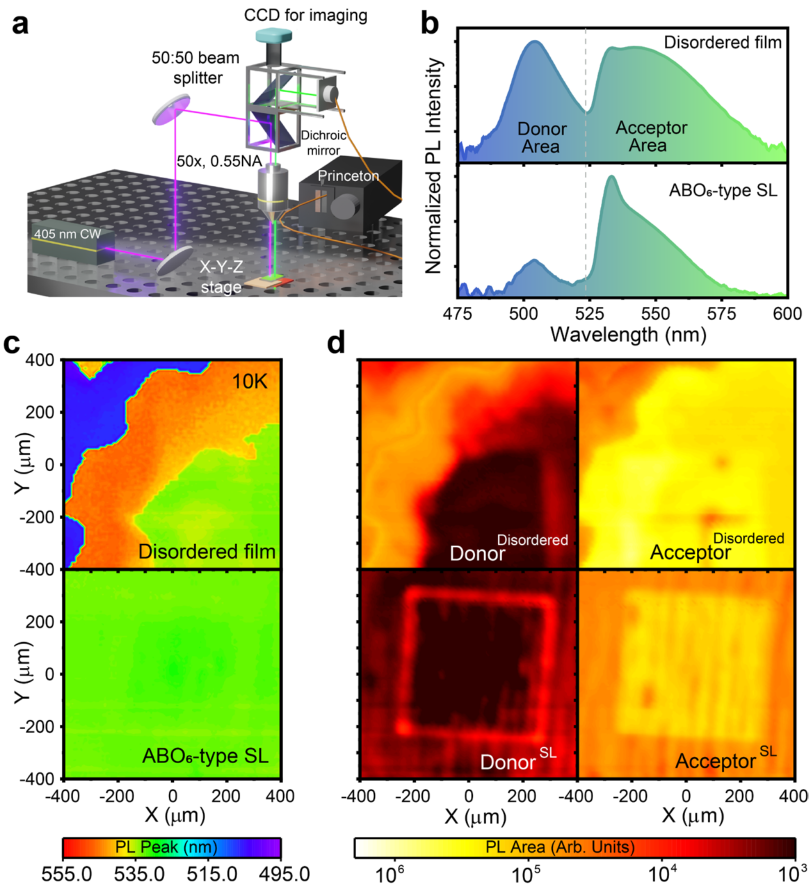

Figure S14. **Hyperspectral PL mapping experiments at cryogenic temperature (10 K), performed on a ABO_6-type binary SL and a disordered film comprising 5.3 nm and 17.6 nm NCs.** (a) Schematic of the custom-made confocal PL mapping setup. (b) PL spectra from different probed areas across the two samples. In parts of the disordered film, strong emission from 5.3 nm NCs is observed as a result of partial segregation of small and large NCs within the film. On the contrary, very similar PL spectral characteristics, characterized by quenched 5.3 nm NC emission and strong 17.6 nm NC luminescence were obtained across the whole surface of the SL sample. The significantly more uniform distribution of the emission and energy in the SL sample is further evidenced by hyperspectral maps of the two samples, plotting: (c) PL peak wavelength and (d) PL intensity at the emission region of the 5.3 nm (donor) and 17.6 nm (acceptor) NCs.

References

(1) Protesescu, L.; Yakunin, S.; Bodnarchuk, M. I.; Krieg, F.; Caputo, R.; Hendon, C. H.; Yang, R. X.; Walsh, A.; Kovalenko, M. V. Nanocrystals of Cesium Lead Halide Perovskites ($CsPbX_3$, X = Cl, Br, and I): Novel Optoelectronic Materials Showing Bright Emission with Wide Color Gamut. *Nano Lett.* **2015**, *15*, 3692-3696.

(2) Dong, Y.; Qiao, T.; Kim, D.; Parobek, D.; Rossi, D.; Son, D. H. Precise Control of Quantum Confinement in Cesium Lead Halide Perovskite Quantum Dots via Thermodynamic Equilibrium. *Nano Lett.* **2018**, *18*, 3716-3722.

(3) Boehme, S. C.; Bodnarchuk, M. I.; Burian, M.; Bertolotti, F.; Cherniukh, I.; Bernasconi, C.; Zhu, C.; Erni, R.; Amenitsch, H.; Naumenko, D.; et al. Strongly Confined $CsPbBr_3$ Quantum Dots as Quantum Emitters and Building Blocks for Rhombic Superlattices. *ACS Nano* **2023**, *17*, 2089-2100.

(4) Bodnarchuk, M. I.; Boehme, S. C.; Ten Brinck, S.; Bernasconi, C.; Shynkarenko, Y.; Krieg, F.; Widmer, R.; Aeschlimann, B.; Gunther, D.; Kovalenko, M. V.; et al. Rationalizing and Controlling the Surface Structure and Electronic Passivation of Cesium Lead Halide Nanocrystals. *ACS Energy Lett.* **2019**, *4*, 63-74.

(5) Bera, S.; Behera, R. K.; Pradhan, N. α-Halo Ketone for Polyhedral Perovskite Nanocrystals: Evolutions, Shape Conversions, Ligand Chemistry, and Self-Assembly. *J. Am. Chem. Soc.* **2020**, *142*, 20865-20874.

(6) Cherniukh, I.; Sekh, T. V.; Raino, G.; Ashton, O. J.; Burian, M.; Travesset, A.; Athanasiou, M.; Manoli, A.; John, R. A.; Svyrydenko, M.; et al. Structural Diversity in Multicomponent Nanocrystal Superlattices Comprising Lead Halide Perovskite Nanocubes. *ACS Nano* **2022**, *16*, 7210-7232.
